# Genome-Wide Analysis of Late Embryogenesis Abundant Protein Gene Family in *Vigna* Species and Expression of *VrLEA* Encoding Genes in *Vigna glabrescens* Reveal Its Role in Heat Tolerance

**DOI:** 10.3389/fpls.2022.843107

**Published:** 2022-03-22

**Authors:** Chandra Mohan Singh, Mukul Kumar, Aditya Pratap, Anupam Tripathi, Smita Singh, Anuj Mishra, Hitesh Kumar, Ramkrishnan M. Nair, Narendra Pratap Singh

**Affiliations:** ^1^Department of Genetics and Plant Breeding, Banda University of Agriculture and Technology, Banda, India; ^2^ICAR-Indian Institute of Pulses Research, Kanpur, India; ^3^World Vegetable Center South Asia, Hyderabad, India

**Keywords:** abiotic stress, candidate genes, expression analysis, heat stress, mung bean, wild *Vigna*, *LEA* genes

## Abstract

Late embryogenesis abundant (LEA) proteins are identified in many crops for their response and role in adaptation to various abiotic stresses, such as drought, salinity, and temperature. The *LEA* genes have been studied systematically in several crops but not in *Vigna* crops. In this study, we reported the first comprehensive analysis of the *LEA* gene family in three legume species, namely, mung bean (*Vigna radiata*), adzuki bean (*Vigna angularis*), and cowpea (*Vigna unguiculata*), and the cross-species expression of *VrLEA* genes in a wild tetraploid species, *Vigna glabrescens*. A total of 201 *LEA* genes from three *Vigna* crops were identified harboring the LEA conserved motif. Among these 55, 64, and 82 *LEA* genes were identified in mung bean, adzuki bean, and cowpea genomes, respectively. These *LEA* genes were grouped into eight different classes. Our analysis revealed that the cowpea genome comprised all eight classes of *LEA* genes, whereas the LEA-6 class was absent in the mung bean genome. Similarly, LEA-5 and LEA-6 were absent in the adzuki bean genome. The analysis of *LEA* genes provides an insight into their structural and functional diversity in the *Vigna* genome. The genes, such as *VrLEA-2*, *VrLEA-40*, *VrLEA-47*, and *VrLEA-55*, were significantly upregulated in the heat-tolerant genotype under stress conditions indicating the basis of heat tolerance. The successful amplification and expression of *VrLEA* genes in *V. glabrescens* indicated the utility of the developed markers in mung bean improvement. The results of this study increase our understanding of *LEA* genes and provide robust candidate genes for future functional investigations and a basis for improving heat stress tolerance in *Vigna* crops.

## Introduction

Mung bean (*Vigna radiata*) is an important short-duration pulse crop occupying a large area, predominantly in the Indian subcontinent, besides many other places in Australia, Africa, and United States. It is primarily consumed as food and animal feed and also as a green manuring crop (Pratap et al., 2021). It has a high nutrition value (Pratap et al., 2020) with a prominence of quality protein besides having soil ameliorative properties (Allito et al., 2015; Sehrawat et al., 2015; Singh et al., 2017). Although several high-yielding and superior varieties have been developed over the last few decades in the mung bean due to their climate sensitivity, improving their productivity in extreme environments is still a major challenge (Singh et al., 2014, 2019a; Manivannan and Mahalingam, 2021). In legume species, such as *Vigna* crops, seed yield is highly affected by various abiotic stresses (HanumanthaRao et al., 2016; Douglas et al., 2020). Drought and high temperature are the most limiting factors (Douglas et al., 2020; Kumar et al., 2020; Singh et al., 2021) among the abiotic stresses, affecting the crops at different growth stages, leading to severe yield penalties. Varieties respond differentially to abiotic stresses as per stress duration, crop growth stage, and genetic potential of a variety, which leads to moderate-to-severe yield loss (Bangar et al., 2019). Hence, there is an urgent need to develop abiotic stress-tolerant varieties to mitigate the effects of climate change.

The heat stress negatively affects the seedling vigor, biomass accumulation, and reproductive development in mung bean (Tzudir et al., 2014; HanumanthaRao et al., 2016). It also affects all reproductive traits, such as flower initiation, pollen viability, stigma receptivity, ovule size and viability, fertilization, pod set, grain filling, and seed quality (Barnabas et al., 2008; Wassmann et al., 2009; Douglas et al., 2020). Flower shedding is very common in mung bean under heat stress (Kumari and Varma, 1983) while it severely affects flower bud initiation, and this sensitivity may prevail for 10–15 days (Bita and Gerats, 2013). Terminal heat stress is a major problem in spring- and summer-grown crops, whereas heat stress during early vegetative growth is frequent in *Kharif* (rainy) season.

A few potential donors have been identified and characterized among the cultivated germplasm and wild relatives in earlier studies against various abiotic stresses (Pratap et al., 2014; Sharma et al., 2016; Singh et al., 2021), which can be included in breeding programs for mung bean improvement. Pratap et al. (2014) characterized two wild accessions belonging to *Vigna umbellata* and *Vigna glabrescens* for photo- and thermoperiod insensitivity. In this study, we investigated the gene expression of *VrLEA* genes in heat-sensitive (HS) mung bean and heat-tolerant (HT) creole bean (*V. glabrescens*) genotypes for understanding the response of selected candidate genes under high-temperature stress. Wild species can be especially useful toward integrating crossable donors in breeding programs and help in improving stress tolerance in domesticated cultivars and broadening the genetic base of the crop.

Abiotic stresses decline crop productivity and seed quality by serious disruptions in plant growth and development (Xiong and Zhu, 2002; Ahuja et al., 2010). Therefore, to defend these adverse climatic conditions, plants have evolved complex regulatory mechanisms at molecular, physiological, and biochemical levels to minimize the effects of abiotic stresses (Zhu, 2016). Proteins play a major role in regulating the various signaling pathways that activate under abiotic stresses and indirectly improve stress tolerance. The late embryogenesis abundant (LEA) protein gene family is considered as an important group of functional proteins to reduce the scope of abiotic stresses in plants (Hirayama and Shinozaki, 2010; Debnath et al., 2011). These proteins can eliminate the cellular content of reactive oxygen species (ROS) to protect the macromolecular substances and alleviate damages caused by the various abiotic stresses (Yu et al., 2019).

The LEA genes were first identified in mature cotton seeds (Dure et al., 1981), which are found to be accumulated during dehydration and maturation of seeds, and protect them from damage. They have also been detected in various plant parts, such as seedlings, leaves, stems, roots, and other organs, under abiotic stress (Hincha and Thalhammer, 2012). Later, LEA proteins have also been identified in other plants, such as *Arabidopsis* (Finkelstein, 1993; Hundertmark and Hincha, 2008), *Brassica napus* (Liang et al., 2016), maize (Li and Cao, 2016), rice (Wang et al., 2007), soybean (Liu et al., 2018), wheat (Liu et al., 2019), grape wine (İbrahime et al., 2019), poplar (Cheng et al., 2021), and many more species. LEA proteins are highly hydrophilic and intrinsically unstructured, whereas they partially fold into mainly α-helical structures under dehydration state (Hand et al., 2011). This allows them to contribute the function as chaperones (Hanin et al., 2011) and also in the stabilization of membranes, calcium-binding, and metal homeostasis and the protection of functional proteins against aggregation (Krueger et al., 2002; Tolleter et al., 2010; Rahman et al., 2011; Rosales et al., 2014). LEA proteins are divided into eight different clusters, including LEA-1, LEA-2, LEA-3, LEA-4, LEA-5, LEA-6, dehydrin (DHN), and seed maturation protein (SMP) (Hundertmark and Hincha, 2008; Finn et al., 2010; Hunault and Jaspard, 2010).

While the LEA gene family also plays an important role in growth and development under multiple stress conditions, the study of this gene family in *Vigna* is still lacking. Many *Vigna* species are now sequenced, such as mung bean (Kang et al., 2014), adzuki bean (Kang et al., 2015), cowpea (Lonardi et al., 2019), beach pea (Singh et al., 2019b), moth bean (Takahashi et al., 2019), and black gram (Souframanien et al., 2020), with their sequence data available in the public database and can be accessed for analysis. Keeping this in view, this experiment was conducted to survey LEA genes and study their comparative structural diversity analysis in mung bean, adzuki bean, and cowpea. The expression of selected *VrLEA* genes through quantitative real-time PCR (qRT-PCR) was performed on HS mung bean and HT creole bean genotypes. It provides new insights for understanding the mechanism of heat tolerance in *Vigna* species.

## Materials and Methods

### Plant Materials and Stress Treatment

The plant materials comprised a high-yielding and HS-advanced breeding line of mung bean, IPM 312-19, and one HT-wild creole bean (*V. glabrescens*), accession IC251372. The plants of both the genotypes were grown in the net house during *Kharif* (rainy season) 2021 at Banda University of Agriculture and Technology, Banda, located at 24°53′ latitude and 25°55′ latitude. Both the genotypes were subjected to heat-shock treatment through the detached leaf method following the study by Kumar et al. (2009) with modification as well as *in planta*.

#### Experiment 1

At the early vegetative stage (20 days after germination), the expanded second trifoliate leaves from the top of the plants were subjected to heat stress treatment through the detached leaf method. This was performed in a growth chamber. The leaves from both the test genotypes (IPM 312-19 and IC 251372) were plucked and kept in Petri plates in distilled water, and heat shock was induced at two levels, namely, HSI 1: 45°C for 3 h with 60% relative humidity (RH); and HSI 2: 45°C for 6 h with 60% RH. For the control treatment (non-stressed), the detached trifoliate leaves were kept in Petri plates in distilled water and put into the growth chamber for 6 h at 25°C temperature with 60% RH to recover them from the injury induced by plucking. Immediately after the heat shock, the stressed and control (non-stressed) samples were taken out, blot-dried by slightly pressing between two layers of filter paper, frozen in liquid nitrogen, and stored at −80°C for further study.

#### Experiment 2

Another experiment was performed *in planta* under laboratory conditions in a plant growth chamber. The plants of both the genotypes were raised in a potting mixture in plastic pots with ideal conditions, such as 30°C temperature with 60% RH. The 7-day-old seedlings were subjected to heat stress. The leaf samples without induction of heat stress were kept as control (non-stress) while those taken 24 h after heat stress (45°C, 60% RH) were considered as stressed plants. The leaf samples from the stressed plants were immediately frozen in liquid nitrogen after plucking at different time points and stored at −80°C for further study.

### RNA Extraction and cDNA Synthesis

A total of 200 mg of frozen leaf samples were homogenized in liquid nitrogen, and RNA was extracted using a Plant RNA Extraction Kit (RNeasy Mini Kit, QIAGEN) following the manufacturer’s instructions. Subsequently, RNA was reverse-transcribed from 1 μg of RNA by the RevertAid First-Strand cDNA Synthesis Kit (Thermo Fisher Scientific). The cDNA was normalized by 50 ng/μl for qRT-PCR analysis.

### Identification of Late Embryogenesis Abundant Protein Sequences

The genome-wide data of mung bean (*V. radiata*), adzuki bean (*Vigna angularis*), and cowpea (*Vigna unguiculata*) were obtained from Legume Information System (LIS) online website^1^. The typical LEA protein genes were retrieved through Pfam analysis with PF03760, PF03168, PF03242, PF02987, PF0477, PF10714, PF0492, and PF00257^2^. The redundancy of sequences was eliminated by ExPASy^3^ to generate a unique set of LEA proteins. The LEA proteins were scanned against the mung bean, adzuki bean, and cowpea genomes by using the HMMER 3.0 program^4^ followed by manual verification with the Pfam database^5^. The protein sequences that did not comprise LEA domains were removed and not included in the analysis.

### Chromosomal Distribution and Phylogenetic Analysis of Late Embryogenesis Abundant Genes

The physical positions (in bps) of the *VrLEA, VaLEA*, and *VuLEA* genes were identified in this study, and the length of chromosomes was obtained from the LIS database. These genes from short arm to long arm were mapped onto their corresponding chromosomes in ascending order using MapChart 2.3 (Voorrips, 2002). The LEA genes identified in the scaffold were not assigned on the chromosome. The putative *VrLEA, VaLEA*, and *VuLEA* genes were classified into eight different groups. The phylogenetic trees were constructed with all the putative LEA genes identified in this study. Multiple alignments of the deduced amino acid sequences were performed using ClustalW (Thompson et al., 2002). The phylogenetic tree was constructed through the maximum likelihood (ML) method (Guindon and Gascuel, 2003) provided in the MEGA 7.0 tool (Tamura et al., 2013) based on the Jones-Taylor-Thornton (JTT) matrix-based model. The bootstrap analysis was set with 1,000 replications for the accuracy of the constructed tree.

### Gene Structure Prediction and Protein Analysis of Late Embryogenesis Abundant Genes

The CDS and the genomic DNA sequences of the corresponding *LEA* genes were retrieved by blast analysis on the NCBI database. The gene structures of LEA were determined by comparing the genomic DNA sequences and their corresponding coding sequences using the Gene Structure Display Server version 2.0 (Hu et al., 2015)^6^. The molecular weight (kDa) and isoelectric point (pI) of LEA sequences were predicted with EndMemo^7^ and pI calculator^8^, respectively (Gasteiger et al., 2003). The motifs were analyzed through the MEME program^9^ (Bailey et al., 2009). In motif analysis, the maximum number of motifs was preset to 10 while the optimum width of motifs was set to 6–50 amino acid residues, and other settings were kept as default.

### Gene-Based Primer Designing and Quantitative Real-Time PCR Analysis

All the *VrLEA* candidate genes were blasted against the *V. glabrescens* genome, and the qRT-PCR primers were designed using the Primer-BLAST tool^10^. Six gene-specific qRT-PCR primers from each clade based on their functions were selected for expression analysis. For PCRs, 10 μl 2X SYBR Green qPCR Master Mix, 1 μl of 10 pmol each forward and reverse primers, and 6 μl nuclease-free water were used. The three-step program was run with 10 min: initial denaturation at 96°C, 40 cycles of 45 s; denaturation at 96°C, 45 s; annealing at 58°C, 45 s; and extension at 72°C, followed by melting at the default setting. The *Actin* gene was used as an internal control. The qRT-PCR analysis was carried out using a Rotor-Gene Q-6000 Real-Time PCR machine (QIAGEN). Three biological replicates were taken for expression profiling, and two technical replicates were used for analysis. The relative expression levels of the genes were calculated using the 2^–ΔΔCT^ method (Livak and Schmittgen, 2001).

## Results

### Genome-Wide Identification of *VrLEA*, *VaLEA*, and *VuLEA* Genes

A total of 201 LEA genes were retrieved from three *Vigna* species, namely, mung bean, adzuki bean, and cowpea. A total of 55 non-redundant *LEA-*sequences from mung bean, 64 from adzuki bean, and 82 from cowpea genomes were identified and considered as putative LEA genes (Table 1). However, 96 *MtLEA-*genes were retrieved from the analysis of the *Medicago truncatula* genome. These sequences were classified into eight different groups, namely, LEA-1, LEA-2, LEA-3, LEA-4, LEA-5, LEA-6, SMP, and DHN. LEA genes accounted for 0.20% of all genes present in mung bean (*VrLEA*), 0.24% of genes present in adzuki bean (*VaLEA*), 0.29% of genes present in cowpea (*VuLEA*), and 0.30% of genes present in model legume *Medicago*. Among the eight classes of the LEA gene subfamily, *VrLEA-6* was absent in mung bean, whereas *VaLEA-5* and *VaLEA-6* were found absent in adzuki bean. The maximum proportion of LEA-2 was noticed in all the three *Vigna* species as 38 *VrLEA-2* genes were observed in mung bean, 45 *VaLEA-2* in adzuki bean, 55 *VuLEA-2* in cowpea, and 68 *VrLEA-2* genes in *Medicago*. The average *LEA* genes per Mb were found as 0.12 genes per Mb in mung bean, 0.15 in adzuki bean, 0.13 in cowpea, and 0.23 genes per Mb in *M. truncatula.*

**TABLE 1:** The proportions of late embryogenesis abundant (*LEA*) genes in three *Vigna* and *Medicago truncatula* genomes*.

Description	Mung bean (*V. radiata*)	Adzuki bean (*V. angularis*)	Cowpea (*V. unguiculata*)	*Medicago truncatula*
Total LEA family proteins	55	64	82	96
LEA-1	01	02	05	06
LEA-2	38	45	55	68
LEA-3	06	10	08	05
LEA-4	02	02	03	02
LEA-5	02	00	02	02
LEA-6	00	00	01	02
SMP	05	02	04	06
DHN	01	03	04	05
Total protein-coding genes	26961	26634	28314	31927
*LEA* genes (%)	0.20	0.24	0.29	0.30
Genome size (Mb)	459.27	447.81	607.06	419.47
Average *LEA* genes per Mb	0.12	0.14	0.13	0.23

*Data accessed from the LIS database on May 10, 2021.

### Chromosomal Distribution of *VrLEA*, *VaLEA*, and *VuLEA* Genes

**FIGURE 1:**
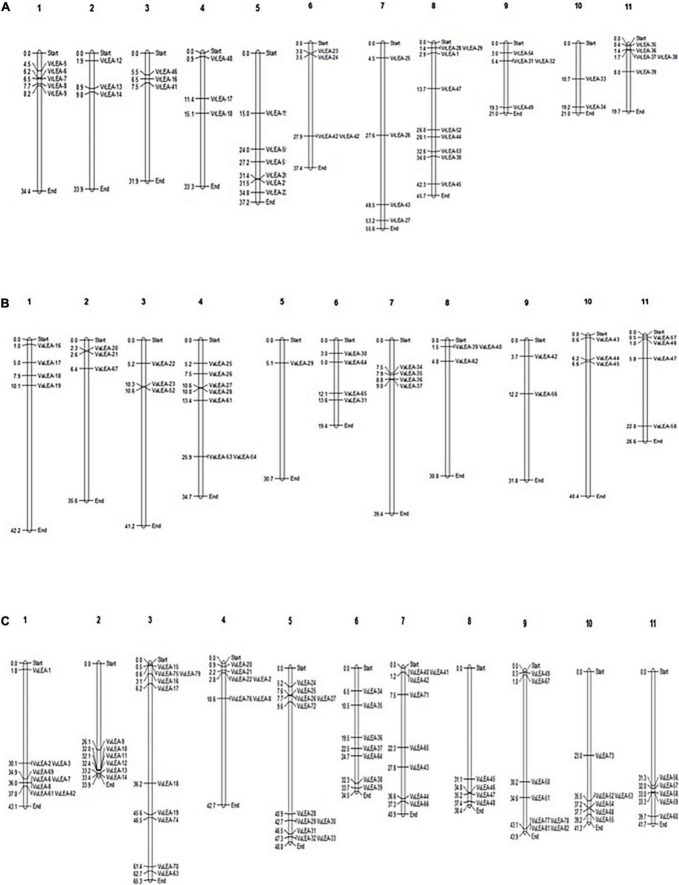
**(A)** Chromosomal distribution of late embryogenesis abundant (*LEA*) genes on mung bean (*Vigna radiata*) genome. **(B)** Chromosomal distribution of *LEA* genes on adzuki bean (*Vigna angularis*) genome. **(C)** Chromosomal distribution of *LEA* genes on cowpea (*Vigna unguiculata*) genome.

The study observed that 48 out of 55 *VrLEA*, 38 out of 62 *VaLEA*, and 80 out of 84 *VuLEA* genes were distributed across 11 chromosomes of mung bean, adzuki bean, and cowpea, respectively (Figure 1), whereas the rest of the *VrLEA*, *VaLEA*, *and VuLEA* genes were present in the scaffold (Supplementary Tables 1–3). Chromosome 10 (ch. 10) had a minimum number of two *VrLEA* genes, whereas ch. 8 had the maximum number of nine *VrLEA* genes in the mung bean genome. Four chromosomes, namely, 1, 2, 10, and 11 had only *VrLEA-2* genes, whereas the rest of the *VrLEA* genes were distributed over seven chromosomes. Similarly, in adzuki bean, ch. 5 comprised only one *VaLEA* gene, whereas ch. 4 had a maximum number of seven *VaLEA* genes. The ch. 1, 7, and 10 are comprised only *VaLEA* genes. In the cowpea genome, ch. 8 had a minimum of four *VuLEA* genes, whereas ch. 5 had a maximum of 11 *VuLEA* genes. Out of 11 chromosomes of cowpea, three chromosomes, namely, ch. 2, 8, and 11 consisted of only *VuLEA* genes while the rest of the 8 chromosomes comprised a mixture of different classes of *VuLEA* genes.

### Phylogenetic Relationship of *VrLEA*, *VaLEA*, and *VuLEA* Genes

**FIGURE 2:**
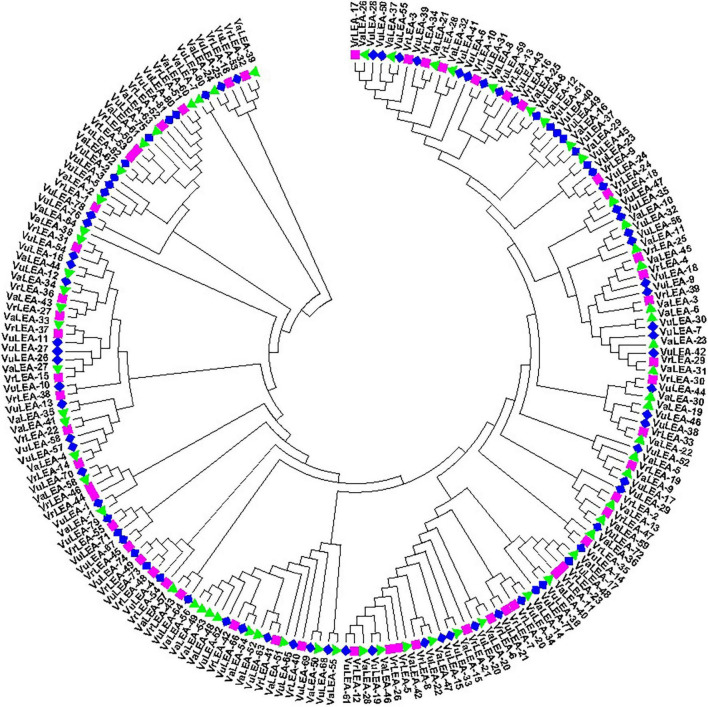
Phylogenetic relationship of *VrLEA*, *VaLEA*, and *VuLEA* genes.

**FIGURE 3:**
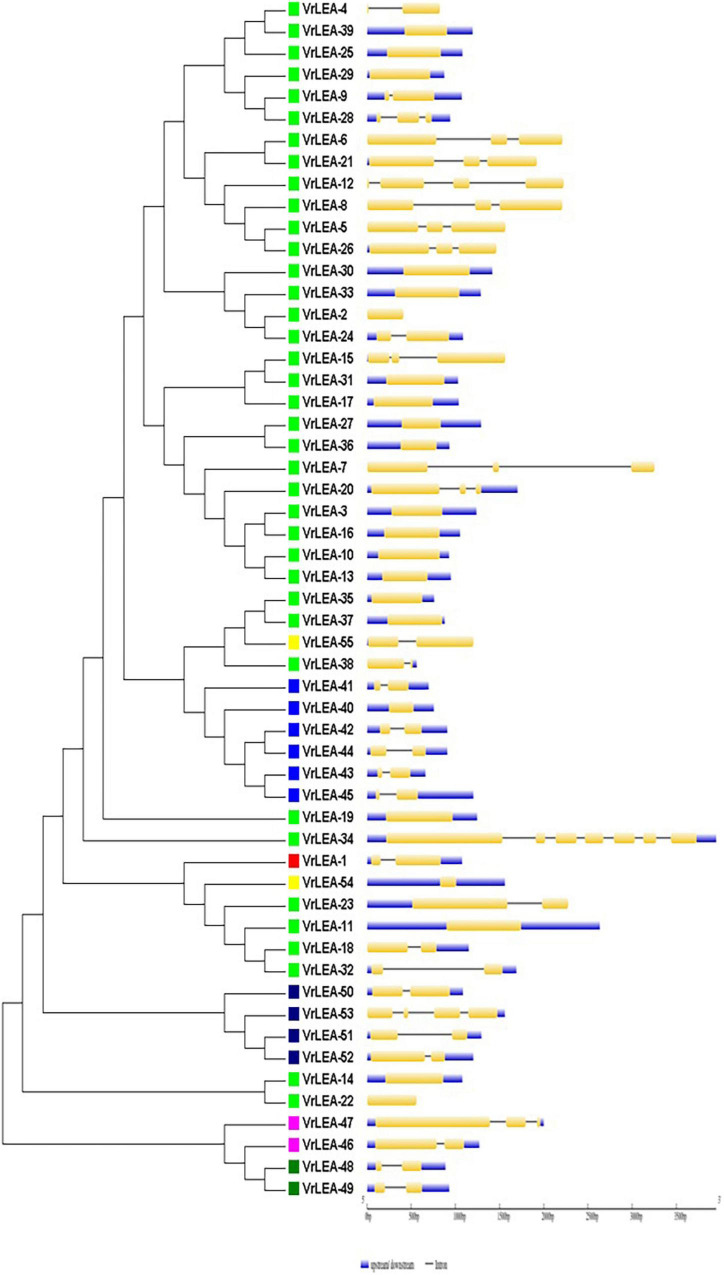
Intron-exon gene structure analysis of *VrLEA* genes.

**FIGURE 4:**
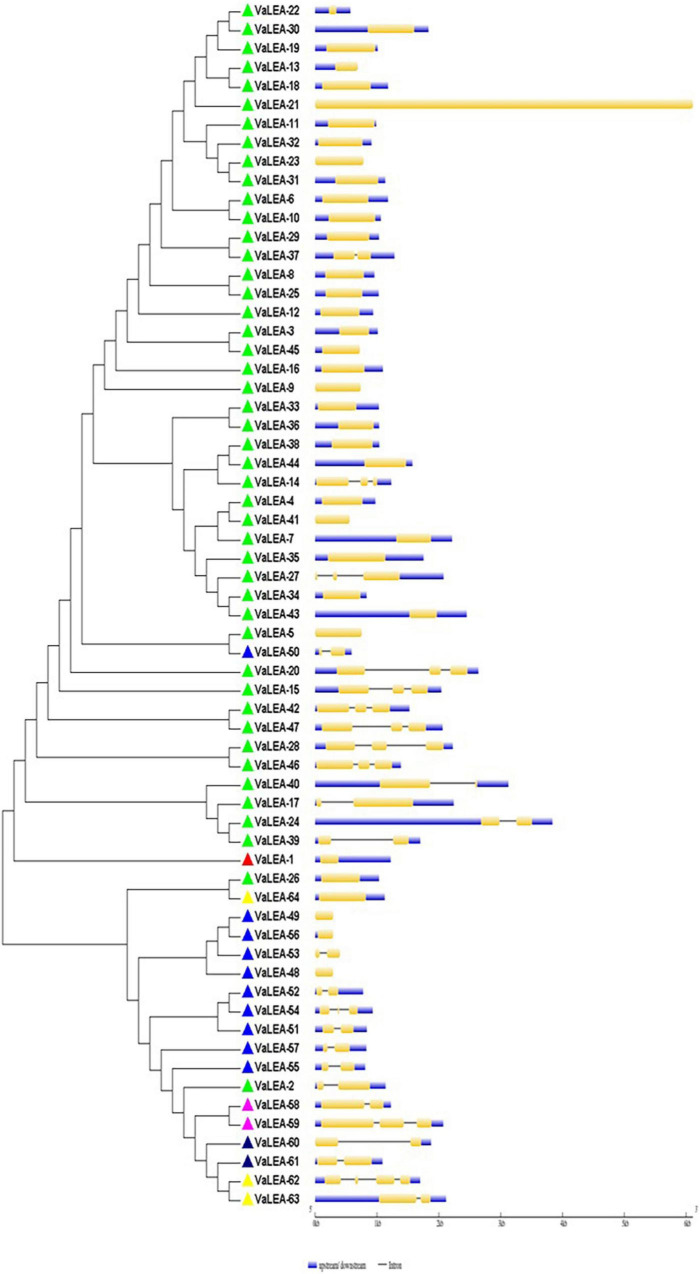
Intron-exon gene structure analysis of *VaLEA* genes.

**FIGURE 5:**
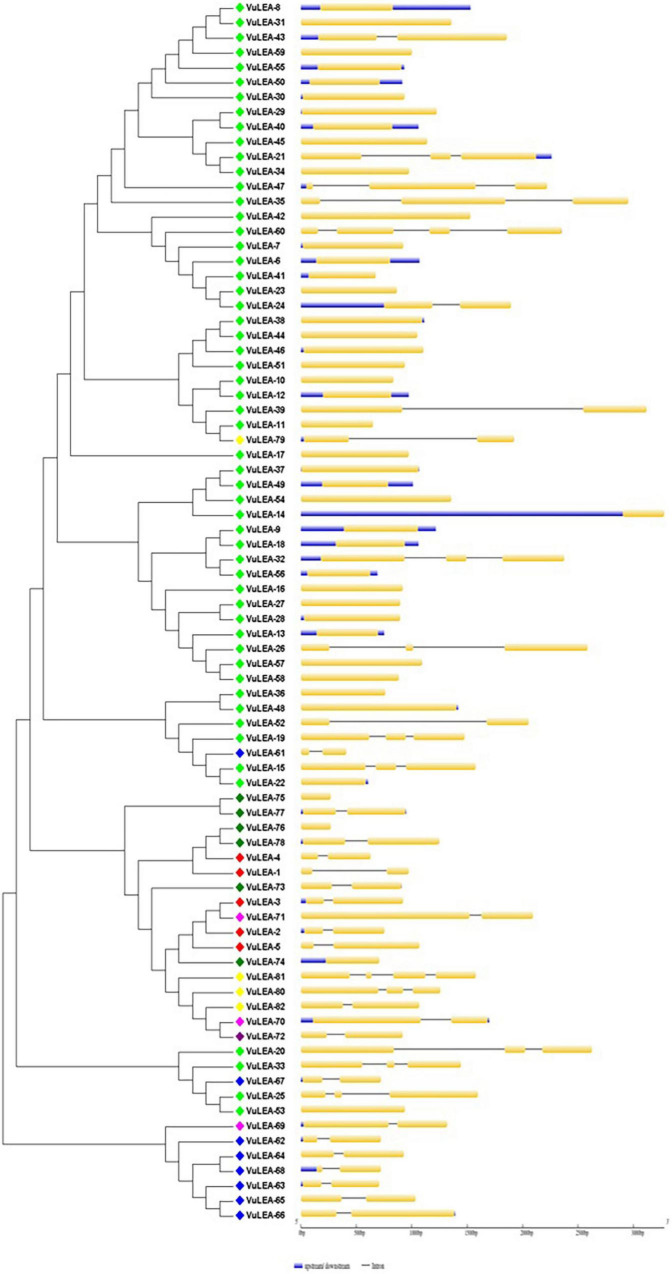
Intron-exon gene structure analysis of *VuLEA* genes.

For the domain-based classification of LEA proteins, all the 201 LEA genes from three *Vigna* species identified in this study were considered for the construction of the phylogenetic tree (Figure 2). The LEA genes verified through Pfam for late embryogenesis-abundant domains were considered as putative *LEA* genes. All of the 201 LEA genes were clearly grouped into 15 major classes indicating their diversity. In mung bean, all the 55 LEA genes were grouped into eight clusters (Figure 3). Clade I comprised 27 *VrLEA*-2 genes, forming the largest cluster. Clade II consisted of 10 *VrLEA* genes, including 3 *VrLEA*-2 genes, 6 *VrLEA*-3 genes, and 1 *VrLEA*-4 gene. Clades III and IV were the single-cluster clades comprising 1 *VrLEA*-2 gene each. Clade V had three types of *LEA* genes, including 1 *VrLEA-*1 and *VrLEA-*4 gene each, and 4 *VrLEA-*2 genes. Clade VI had 4 *VrSMP* genes, and Clade VII had 2 *VrLEA-*2 genes. Similarly, Clade VIII had four *VrLEA* genes, including 2 *VrLEA-*5 and *VrLEA-*6 genes each. The LEA genes in adzuki bean clearly grouped into three major clades (Figure 4). Clade I comprised 44 *VaLEA-*2 genes and 1 *VaLEA-*3 gene. Clade II consisted of only one gene of *VrLEA-*1. Clade III had 2 *VaLEA-2*, 3 *VaDHN*, 9 *VaLEA-*3, 2 *VaLEA-*4, and SMP genes each. Similarly, all the 82 LEA genes from the cowpea genome were grouped into four clades (Figure 5). Clade I comprised 50 *VuLEA-2* genes, and 1 *VuLEA-3* and *VuSMP* gene each whereas Clade II consisted of five different types of *VuLEA* genes. Clade III had 4 *VuLEA-2* genes and 1 *VuLEA-3* gene. Six *VuLEA*-3 genes and 1 *VuLEA-*4 gene were grouped together in Clade IV. This phylogenetic analysis also provided information about diversification and evolutionary relationships among the LEA genes.

### Characterization of *VrLEA*, *VaLEA*, and *VuLEA* Genes

The analyses about the length of LEA protein, genomic and CDS sequence, number of exons, molecular weight, and pI are presented in Supplementary Tables 1–3. The genomic length of *LEA* genes ranged from 282 bp (V*rLEA-40*) to 22,112 bp (*VrLEA-34*) in mung bean, 249 (*VaLEA-56*) to 30,173 bp (*VaLEA-21*) in adzuki bean, and 410 (*VuLEA-61*) to 4711 bp (V*uLEA-70*) in cowpea. Simultaneously, the length of coding sequences varied from 282 bp (*VrLEA-40*) to 7,113 bp (*VrLEA-34*) in mung bean, 249 (*VaLEA-56*) to 3,993 bp (*VaLEA-21*) in adzuki bean, and 270 (*VuLEA-76*) to 2,386 bp (*VuLEA-24*) in cowpea. The number of exons ranged from 1 to 7 in mung bean, whereas it varied from 1 to 4 in adzuki bean and cowpea, respectively. The LEA protein length varied from 94 (*VrLEA-34*) to 2,354 (*VrLEA-34*) in mung bean, 83 (*VrLEA-55*) to 1,050 (*VrLEA-64*) in adzuki bean, and 83 to 1,050 in cowpea. The predicted molecular weight of LEA proteins from mung bean, adzuki bean, and cowpea ranged from 10.22 kDa (*VrLEA-48*) to 263.50 kDa (*VrLEA-34*), 9.36 kDa (*VaLEA-55*) to 1063.50 kDa (*VaLEA-59*), and 21.11 kDa (*VuLEA-76*) to 199.46 kDa (*VuLEA-44*), respectively. Similarly, pI ranged from 4.47 (*VrLEA-50*) to 9.78 (*VrLEA-31*), 4.40 (*VaLEA-6*0) to 11.02 (*VaLEA-54*), and 3.85 (*VuLEA-60*) to 4.37 (*VuLEA-76*) in the three *Vigna* species under study, indicating the structural diversity of LEA genes.

### Gene Structure and Motif Analysis of *VrLEA*, *VaLEA*, and *VuLEA* Genes

**FIGURE 6:**
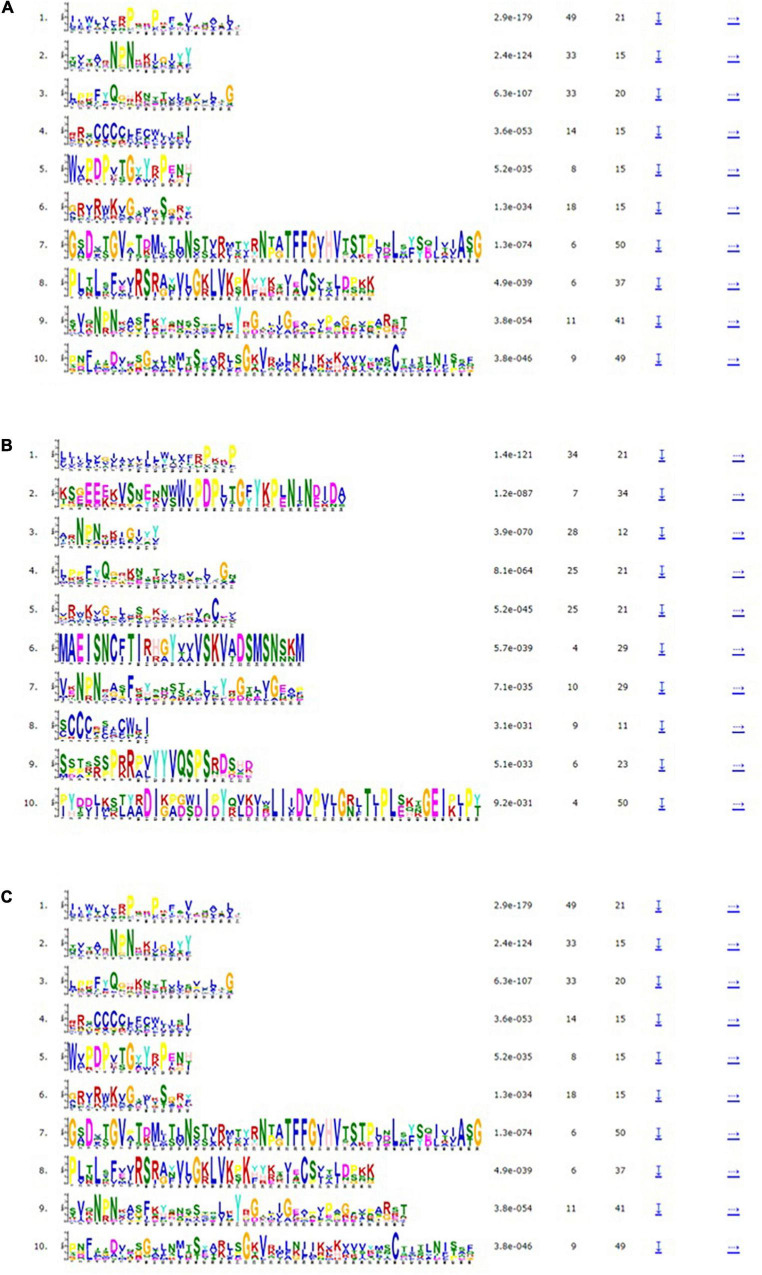
Motif analysis of **(A)** *VrLEA*, **(B)** *VaLEA*, and **(C)** *VuLEA* genes.

The exon-intron organization of the 201 LEA genes from mung bean, adzuki bean, and cowpea was constructed using the coding and genomic sequences (Figures 3–5). The gene structures of LEA genes revealed that all the *LEA* genes exhibited variable exon-intron organization. It ranged from 1 to 7 exons in mung bean and 1 to 4 exons in both adzuki bean and cowpea. The maximum number of introns was found in mung bean as compared to adzuki bean and cowpea, indicating the diversification of the mung bean genome. A total of 10 distinct motifs were identified for LEA genes from mung bean, adzuki bean, and cowpea (Figure 6).

### Expression Pattern of *VrLEA* Genes in Response to Heat Stress

**FIGURE 7:**
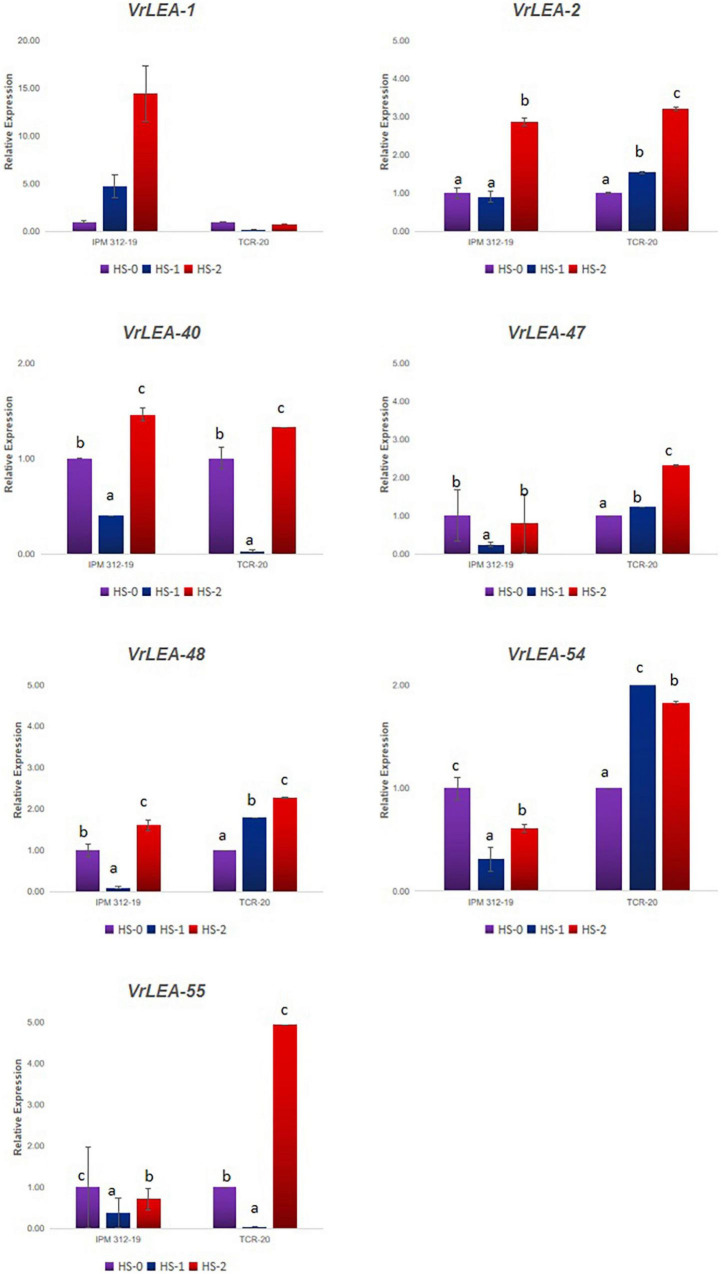
qRT-PCR analysis of selected *VrLEA* candidate genes on HS-mungbean (IPM 312-19) and HR-*V. glabrescens* (IC251372) under heat shock stress through detached leaf method. The statistical test was performed separately in both genotypes. The same letters show non-significant differences, whereas different letters show significant differences.

All the 55 *VrLEA* candidate genes were used for primer designing, and subsequently, 55 primer pairs were developed. These primers were BLAST-searched against the *V. glabrescens* genome, and their annotation was performed (Supplementary Table 4). Seven representative candidates from each subclade were selected for the expression analysis (Figure 7). The expression of these seven genes (*VrLEA-1, VrLEA-2, VrLEA-40, VrLEA-47, VrLEA-48, VrLEA-54*, and *VrLEA-55*) in the HS (IPM 312-19) and HT (IC251372) genotypes was analyzed. The gene *VrLEA-1* exhibited a significant increase (about 15-fold) in HS genotypes on heat shock, whereas a decrease was noticed in the HT genotype. The *VrLEA-2* transcript significantly increased in HS genotype and decreased in HT genotype (IC 251372) at both the levels of heat stress (HSI-1 and HSI-2; 45°C for 3 and 6 h, respectively). The expression of *VrLEA-40* decreased in both the genotypes at HSI-1, whereas significantly increased in both the genotypes at HSI-2. The *VrLEA-47* exhibited a significant decrease in HS genotype, whereas it increased significantly to about 2.5-fold at HSI-2. The pattern of expression of *VrLEA-48* was similar to that of *VrLEA-47*, although the level of expression was higher than *VrLEA-47.* The *VrLEA-54* transcript was downregulated in the HS genotype after the heat sock, whereas it was upregulated significantly to about 2-fold in the HT genotype after the heat-shock induction. Similarly, about a 5-fold increase was noticed in HT genotypes at HSI-2 as compared to control.

**FIGURE 8:**
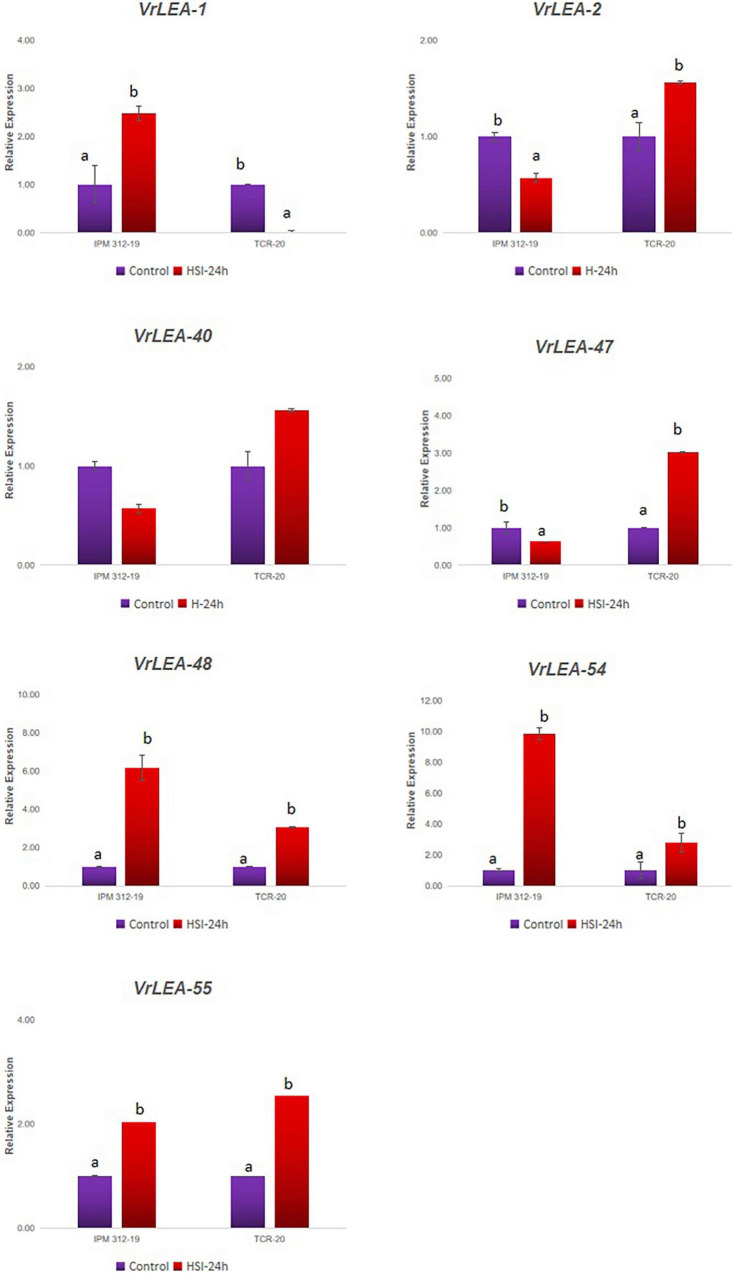
qRT-PCR analysis of selected *VrLEA* candidate genes on HS-mungbean (IPM 312-19) and HR-*V. glabrescens* (IC251372) under heat shock stress at seedling stage. The statistical test was performed separately in both genotypes. The same letters show non-significant differences, whereas different letters show significant differences.

In the second experiment, the 7-day-old seedlings of HS and HT genotypes were subjected to 24-h heat-shock induction and they exhibited differential expression of the genes (Figure 8). The gene *VrLEA-1* exhibited a similar pattern of expression as in the case of the detached leaf method although it was comparatively lower. The expression of three genes, namely, *VrLEA-2, VrLEA-40*, and *VrLEA-47*, was downregulated in the HS genotypes after 24 h of heat stress, whereas it was significantly upregulated in the HT genotype. In contrast, *VrLEA-48* and *VrLEA-54* exhibited upregulation in both HS and HT genotypes, although the level was higher in the HS genotype. Similarly, *VrLEA-55* exhibited upregulation in both HS and HT genotypes with a higher expression in the HT genotype as compared to the HS genotype.

## Discussion

Abiotic stresses encompass extreme environmental factors which alter a plant’s biochemical and physiological processes constraining its productivity (Douglas et al., 2020). Abiotic stresses, either individually or in combination, hinder the crops from reaching their actual genetic yield potential. Among the abiotic stresses, heat stress is one of the most important and frequent constraints that limit the productivity of crop plants, especially during the current scenario of climate change, and is, therefore, a concern for crop cultivation worldwide. The crop yield of mung bean is highly affected by the fluctuating temperatures as the progression and the phenological development of the plant are mainly driven by temperature (Carberry, 2007; Chauhan et al., 2010). While the optimum temperatures for mung bean are 28–30°C (Kaur et al., 2015), the key temperatures are 7.5°C (baseline), 30°C (optimum), and 40°C (maximum). Most of the *Vigna* crops are very frequently subjected to extreme environmental conditions that are many times at the upper extremes as far as temperature is concerned. However, a very limited study has been conducted till present on the aspect of high-temperature tolerance in mung bean (Kumar et al., 2016, 2017). The linkage of high-throughput phenotyping platforms for the screening of target traits with omics interventions can give a clue for its mitigation (Pratap et al., 2019). Besides, the genome-wide analysis of gene families and transcriptional factors helps in designing crops for improved heat tolerance in mung bean.

The LEA gene family is large and widely diversified across the plant kingdom (Gao and Lan, 2016). It is an important gene family that responds to plant developmental activities, biotic and abiotic stresses (Hincha and Thalhammer, 2012), and hormonal regulations (Liu et al., 2019). This gene family has been characterized in many crops, such as *Arabidopsis* (Hundertmark and Hincha, 2008), wheat (Liu et al., 2019), tea (Jin et al., 2019), potato (Chen et al., 2019), and poplar (Cheng et al., 2021). Besides, this gene family was also identified in fungi and bacteria (Garay-Arroyo et al., 2000; Gal et al., 2004; Gao et al., 2016). In contrast, the genome-wide identification and characterization of the LEA gene family in mung bean, adzuki bean, and cowpea have not yet been performed systematically. In this study, a total of 201 LEA genes were identified, in which 55, 64, and 82 *LEA* genes were found in mung bean, adzuki bean, and cowpea genomes, respectively. The analysis revealed that these genes belong to six to eight classes of the LEA gene family. Based on the conserved domain and phylogenetic tree analyses, 55 *VrLEA* genes were categorized in seven distinct groups, 64 *VaLEA* genes in six groups, and 82 *VuLEA* genes in eight groups. The cowpea genome had all the eight types of LEA subfamilies whereas the adzuki bean genome lacked two subfamilies of *VaLEA* genes (i.e., *VaLEA*-5 and *VaLEA-*6), and the mung bean genome lacked the *VrLEA-*6 subfamily. It was indicated that LEA-1 to LEA-4, SMP, and DHN were highly conserved. The LEA-6 group has also been found to be absent in a few higher plants, such as *Dendrobium officinale* (Ling et al., 2016) and *Solanum lycopersicum* (Cao and Li, 2015). This finding indicated that the variation exists in the LEA protein gene family groups in some of the plant species. In the three *Vigna* species studied, the maximum proportion of the LEA gene family was accounted for LEA-2 as compared to other LEA groups. A similar type of contribution of LEA-2 has been observed earlier by Jin et al. (2019) in tea. In contrast, a similar proportion of the LEA-2 group was not observed earlier in many plant species, such as *Arabidopsis thaliana* (Hundertmark and Hincha, 2008), *Cleistogenes songorica* (Muvunyi et al., 2018), *Manihot esculenta* (Wu et al., 2018), *Pinus tabuliformis* (Gao et al., 2016), and *Populus trichocarpa* (Lan et al., 2013). This difference may be attributed to the improvement in the annotation of genomes of plants species. The differences were observed in all the groupings of *VrLEA, VaLEA, and VuLEA* genes in the phylogenetic analysis, indicating their diversification and differential genetic pattern. This finding also indicated the variation and complexity of the LEA gene family in plants. The gene structure prediction of LEA genes revealed that most of the LEA genes contained only a few or no introns, and all encoded the conserved LEA domain.

**FIGURE 9:**
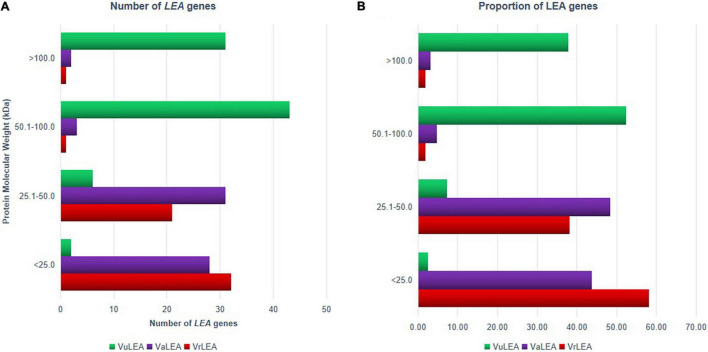
**(A)** Number and **(B)** proportion of LEA protein in *VrLEA*, *VaLEA*, and *VuLEA* gene families based on their molecular weight.

Based on the physiochemical properties of LEA genes, it was noticed that most of the *LEA* genes (58.18%) encode relatively small proteins (<25 kDa) in the mung bean genome (Figure 9A), whereas only 3.60% of them are large proteins (>50 kDa) (Figure 9B). Similarly, about 43.75% of genes encode small proteins in the adzuki bean genome and 7.81% of them encode larger proteins. On the contrary, only 2.44% of genes are <25 kDa in the cowpea genome, whereas most of the genes (about 52.44%) had a high molecular weight (50.1–100 kDa). This result was in conformity with the findings of Chen et al. (2019) in *Solanum tuberosum* (94.6%) and those of Liang et al. (2016) in *B. napus* (90.7%). Therefore, while this report is in conformity with the earlier reports in the case of mung bean and adzuki bean, it is contrasting in the case of the cowpea genome. The analysis also revealed that each LEA group contained conserved motifs that have been previously reported in other plant species (Liang et al., 2016; Magwanga et al., 2018). This was indicated that *LEA* genes may encode functional LEA proteins that have specific functions within the group, and the members of the same protein within the group may have originated from gene expansion. The members of the different groups may be due to the evolution from different ancestors.

Most of the LEA proteins are predicted to have no stable secondary structure in solution, but they may acquire α-helical structures on dehydration (Bremer et al., 2017), which allows them to conformational change according to the changes in their microenvironment, resulting in multiple functions in abiotic stresses (Hanin et al., 2011). Previous studies have shown that *LEA* genes play an important role in low-temperature, drought, and high-salinity stresses (Jin et al., 2019). The expression of LEA proteins is often induced by abiotic stresses, such as drought, temperature, salt, and exogenous application of hormones, at different development stages and tissues of plants (Chen et al., 2019), and an increased expression of the *LEA* genes can improve stress tolerance. The overexpression of the wheat *DHN-5* was reported to enhance osmotic stress tolerance in *Arabidopsis* (Brini et al., 2011). Ding et al. (2021) reported the maximum expression of *LEA* genes under polyethylene glycol (PEG) treatment, low temperature, and light intensity deviations in the case of rye. Therefore, the above-mentioned studies demonstrate that the LEA genes could potentially be used to improve the abiotic stress tolerance of crops. The expression analysis of HS and HT genotypes in this study indicated that the genes *VrLEA-1, VrLEA-47, VrLEA-48, VrLEA-54*, and *VrLEA-55* play an important role toward heat stress tolerance in the HT-wild *Vigna*, accession IC 251372. Similarly, *LEA* genes, such as *VrLEA-2, VrLEA-40, VrLEA-47*, and *VrLEA-55*, were significantly overexpressed in the HT genotype, indicating the basis of heat tolerance in IC251372. The *VrLEA-55* (DHN protein) gene showed a 5-fold increase in the expression in HT genotype in Experiment-1 (detached leaf method), whereas about two-fold increase was noticed at the seedling stage under 24 h of heat induction in the intact seedling (*in planta* heat stress treatment). The differential expression of the *VrLEA-55* gene in both treatments could be attributed to mechanical injury in addition to heat stress in the detached leaf method as compared to the *in planta* method. Ding et al. (2021) also noticed the expression of this gene in different tissues, including seeds of rye, in drought stress although the expression was non-significant. Our study reports the expression of this candidate gene in the leaves of the HT-wild *Vigna* genotype and provides a new insight to heat tolerance. Therefore, the results of this study laid the foundation for further investigating the functional characterization of LEA proteins and their potential use in the genetic improvement of mung bean for heat tolerance.

## Conclusion

A total of 201 LEA genes were identified in three *Vigna* species which were further grouped into seven distinct clades based on their phylogenetic relationships. The LEA-6 group was found absent in the mung bean genome, whereas all the seven groups were present in the cowpea genome. The gene structure exhibited diversification of candidates in the *Vigna* genome. Additionally, the expression profiling of seven *VrLEA* genes in leaves under four heat stress treatments revealed that these genes had an important role in response to heat stress. These results, therefore, provide valuable information for the future functional studies of *LEA* genes toward the genetic improvement of mung bean for heat stress tolerance.

## Data Availability Statement

The datasets presented in this study can be found in online repositories. The names of the repository/repositories and accession number(s) can be found in the article/Supplementary Material.

## Author Contributions

CMS, MK, and AP conceived the idea and planned the work. SS, AT, AM, and HK retrieved the data from the database. CMS and AP analyzed the data. CMS drafted the manuscript. AP, RN, and NS edited the manuscript. All authors read the manuscript and approved it for publication.

## Conflict of Interest

The authors declare that the research was conducted in the absence of any commercial or financial relationships that could be construed as a potential conflict of interest.

## Publisher’s Note

All claims expressed in this article are solely those of the authors and do not necessarily represent those of their affiliated organizations, or those of the publisher, the editors and the reviewers. Any product that may be evaluated in this article, or claim that may be made by its manufacturer, is not guaranteed or endorsed by the publisher.

## Footnotes

1
https://legumeinfo.org/

2
https://legumeinfo.org/search/gene

3
https://web.expasy.org/decrease_redundancy/

4
https://www.ebi.ac.uk/Tools/hmmer/

5
http://pfam.xfam.org/

6
http://gsds.gao-lab.org/

7
http://www.endmemo.com/bio/promw.php

8
http://isoelectric.org/

9
https://meme-suite.org/meme/tools/meme

10
https://www.ncbi.nlm.nih.gov/tools/primer-blast/
