# Supplementary material for: Genome-Wide Analysis of Late Embryogenesis Abundant Protein Gene Family in Vigna Species and Expression of VrLEA Encoding Genes in Vigna glabrescens Reveal Its Role in Heat Tolerance
Source: Front Plant Sci. 2022 Mar 22;13:843107. doi: 10.3389/fpls.2022.843107 (PMC8981728; doi:10.3389/fpls.2022.843107)
Supplement: Supplementary file 1 [file Table_1.DOCX]

**Supplementary Table S1.** The *VrLEA* candidates identified in mungbean genome and their gene characteristics

| **Alias Name** | **Gene ID** | **Family** | **LG** | **Gene Start (bps)** | **Gene End (bps)** | **Genomic Length** | **CDS Length** | **Number of Exon** | **Protein length (aa)** | **Protein Mol. Wt (kDa)** | **pI** |
| --- | --- | --- | --- | --- | --- | --- | --- | --- | --- | --- | --- |
| VrLEA-1 | [vigra.Vradi08g01880](https://legumeinfo.org/feature/Vigna/radiata/gene/Vradi08g01880.Vradi.ver6) | LEA-1 | 8 | 2923800 | 2924589 | 789 | 624 | 2 | 208 | 21.29 | 4.97 |
| VrLEA-2 | [vigra.Vradi0086s00270](https://legumeinfo.org/feature/Vigna/radiata/gene/Vradi0086s00270.Vradi.ver6) | LEA-2 | vigra.scaffold_86 | 434761 | 435175 | 414 | 414 | 1 | 138 | 15.57 | 5.41 |
| VrLEA-3 | [vigra.Vradi0179s00410](https://legumeinfo.org/feature/Vigna/radiata/gene/Vradi0179s00410.Vradi.ver6) | LEA-2 | vigra.scaffold_179 | 8652 | 11750 | 3098 | 702 | 1 | 234 | 25.48 | 7.87 |
| VrLEA-4 | [vigra.Vradi0180s00130](https://legumeinfo.org/feature/Vigna/radiata/gene/Vradi0180s00130.Vradi.ver6) | LEA-2 | vigra.scaffold_180 | 327317 | 328143 | 826 | 441 | 1 | 147 | 16.72 | 4.89 |
| VrLEA-5 | [vigra.Vradi01g02230](https://legumeinfo.org/feature/Vigna/radiata/gene/Vradi01g02230.Vradi.ver6) | LEA-2 | 1 | 4527323 | 4528992 | 1669 | 1483 | 3 | 299 | 33.33 | 9.68 |
| VrLEA-6 | [vigra.Vradi01g03900](https://legumeinfo.org/feature/Vigna/radiata/gene/Vradi01g03900.Vradi.ver6) | LEA-2 | 1 | 6166844 | 6169174 | 2330 | 1585 | 3 | 305 | 33.24 | 8.98 |
| VrLEA-7 | [vigra.Vradi01g04160](https://legumeinfo.org/feature/Vigna/radiata/gene/Vradi01g04160.Vradi.ver6) | LEA-2 | 1 | 6465992 | 6469571 | 3579 | 1350 | 3 | 246 | 27.53 | 7.69 |
| VrLEA-8 | [vigra.Vradi01g04920](https://legumeinfo.org/feature/Vigna/radiata/gene/Vradi01g04920.Vradi.ver6) | LEA-2 | 1 | 7658352 | 7660658 | 2306 | 1519 | 3 | 298 | 32.89 | 9.43 |
| VrLEA-9 | [vigra.Vradi01g05310](https://legumeinfo.org/feature/Vigna/radiata/gene/Vradi01g05310.Vradi.ver6) | LEA-2 | 1 | 8150504 | 8160405 | 9901 | 927 | 2 | 309 | 35.62 | 9.51 |
| VrLEA-10 | [vigra.Vradi0234s00370](https://legumeinfo.org/feature/Vigna/radiata/gene/Vradi0234s00370.Vradi.ver6) | LEA-2 | vigra.scaffold_234 | 47686 | 57006 | 9320 | 1269 | 1 | 423 | 46.45 | 9.25 |
| VrLEA-11 | [vigra.Vradi0262s00060](https://legumeinfo.org/feature/Vigna/radiata/gene/Vradi0262s00060.Vradi.ver6) | LEA-2 | vigra.scaffold_262 | 39126 | 41299 | 2173 | 903 | 1 | 301 | 33.3 | 4.93 |
| VrLEA-12 | [vigra.Vradi02g02030](https://legumeinfo.org/feature/Vigna/radiata/gene/Vradi02g02030.Vradi.ver6) | LEA-2 | 2 | 1931302 | 1933598 | 2296 | 1213 | 4 | 316 | 35.73 | 9.51 |
| VrLEA-13 | [vigra.Vradi02g07400](https://legumeinfo.org/feature/Vigna/radiata/gene/Vradi02g07400.Vradi.ver6) | LEA-2 | 2 | 8925755 | 8927004 | 1249 | 603 | 1 | 201 | 22.56 | 9.63 |
| VrLEA-14 | [vigra.Vradi02g07930](https://legumeinfo.org/feature/Vigna/radiata/gene/Vradi02g07930.Vradi.ver6) | LEA-2 | 2 | 9839173 | 9839830 | 657 | 657 | 1 | 219 | 24.26 | 9.1 |
| VrLEA-15 | [vigra.Vradi0360s00010](https://legumeinfo.org/feature/Vigna/radiata/gene/Vradi0360s00010.Vradi.ver6) | LEA-2 | vigra.scaffold_360 | 220302 | 221872 | 1570 | 1120 | 3 | 193 | 20.77 | 8.82 |
| VrLEA-16 | [vigra.Vradi03g05030](https://legumeinfo.org/feature/Vigna/radiata/gene/Vradi03g05030.Vradi.ver6) | LEA-2 | 3 | 6534677 | 6535307 | 630 | 630 | 1 | 210 | 23.67 | 9.32 |
| VrLEA-17 | [vigra.Vradi04g05100](https://legumeinfo.org/feature/Vigna/radiata/gene/Vradi04g05100.Vradi.ver6) | LEA-2 | 4 | 11398672 | 11399344 | 672 | 672 | 1 | 224 | 24.62 | 8.68 |
| VrLEA-18 | [vigra.Vradi04g07140](https://legumeinfo.org/feature/Vigna/radiata/gene/Vradi04g07140.Vradi.ver6) | LEA-2 | 4 | 15121331 | 15127845 | 6514 | 1117 | 2 | 299 | 32.52 | 4.7 |
| VrLEA-19 | [vigra.Vradi05g07590](https://legumeinfo.org/feature/Vigna/radiata/gene/Vradi05g07590.Vradi.ver6) | LEA-2 | 5 | 15045355 | 15046111 | 756 | 756 | 1 | 252 | 27.67 | 8.8 |
| VrLEA-20 | [vigra.Vradi05g20270](https://legumeinfo.org/feature/Vigna/radiata/gene/Vradi05g20270.Vradi.ver6) | LEA-2 | 5 | 31448691 | 31449935 | 1244 | 908 | 3 | 220 | 24.45 | 9.12 |
| VrLEA-21 | [vigra.Vradi05g20370](https://legumeinfo.org/feature/Vigna/radiata/gene/Vradi05g20370.Vradi.ver6) | LEA-2 | 5 | 31518783 | 31520750 | 1967 | 1554 | 1 | 310 | 34.24 | 9.28 |
| VrLEA-22 | [vigra.Vradi05g22640](https://legumeinfo.org/feature/Vigna/radiata/gene/Vradi05g22640.Vradi.ver6) | LEA-2 | 5 | 34848211 | 34848772 | 561 | 561 | 1 | 187 | 20.66 | 7.18 |
| VrLEA-23 | [vigra.Vradi06g02980](https://legumeinfo.org/feature/Vigna/radiata/gene/Vradi06g02980.Vradi.ver6) | LEA-2 | 5 | 3029945 | 3031789 | 1844 | 1453 | 2 | 315 | 34.85 | 4.62 |
| VrLEA-24 | [vigra.Vradi06g03260](https://legumeinfo.org/feature/Vigna/radiata/gene/Vradi06g03260.Vradi.ver6) | LEA-2 | 6 | 3474872 | 3475694 | 822 | 648 | 2 | 216 | 23.63 | 8.36 |
| VrLEA-25 | [vigra.Vradi07g02640](https://legumeinfo.org/feature/Vigna/radiata/gene/Vradi07g02640.Vradi.ver6) | LEA-2 | 7 | 4539316 | 4539928 | 612 | 612 | 1 | 204 | 22.25 | 8.26 |
| VrLEA-26 | [vigra.Vradi07g10600](https://legumeinfo.org/feature/Vigna/radiata/gene/Vradi07g10600.Vradi.ver6) | LEA-2 | 7 | 27637197 | 27638635 | 1438 | 1290 | 3 | 323 | 35.55 | 9.42 |
| VrLEA-27 | [vigra.Vradi07g29490](https://legumeinfo.org/feature/Vigna/radiata/gene/Vradi07g29490.Vradi.ver6) | LEA-2 | 7 | 53227211 | 53227964 | 753 | 447 | 1 | 149 | 16.05 | 7.52 |
| VrLEA-28 | [vigra.Vradi08g01030](https://legumeinfo.org/feature/Vigna/radiata/gene/Vradi08g01030.Vradi.ver6) | LEA-2 | 8 | 1414975 | 1415603 | 628 | 372 | 3 | 124 | 13.59 | 8.3 |
| VrLEA-29 | [vigra.Vradi08g01040](https://legumeinfo.org/feature/Vigna/radiata/gene/Vradi08g01040.Vradi.ver6) | LEA-2 | 8 | 1439601 | 1440288 | 687 | 687 | 1 | 229 | 26.1 | 8.5 |
| VrLEA-30 | [vigra.Vradi08g14150](https://legumeinfo.org/feature/Vigna/radiata/gene/Vradi08g14150.Vradi.ver6) | LEA-2 | 8 | 33977857 | 33978607 | 750 | 750 | 1 | 250 | 27.79 | 8.87 |
| VrLEA-31 | [vigra.Vradi09g03920](https://legumeinfo.org/feature/Vigna/radiata/gene/Vradi09g03920.Vradi.ver6) | LEA-2 | 9 | 5352475 | 5353135 | 660 | 660 | 1 | 220 | 24.39 | 9.78 |
| VrLEA-32 | [vigra.Vradi09g03950](https://legumeinfo.org/feature/Vigna/radiata/gene/Vradi09g03950.Vradi.ver6) | LEA-2 | 9 | 5360987 | 5362471 | 1484 | 342 | 2 | 114 | 12.69 | 5.16 |
| VrLEA-33 | [vigra.Vradi10g04220](https://legumeinfo.org/feature/Vigna/radiata/gene/Vradi10g04220.Vradi.ver6) | LEA-2 | 10 | 10658322 | 10659054 | 732 | 732 | 1 | 244 | 27.46 | 9.25 |
| VrLEA-34 | [vigra.Vradi10g11640](https://legumeinfo.org/feature/Vigna/radiata/gene/Vradi10g11640.Vradi.ver6) | LEA-2 | 10 | 19204261 | 19226373 | 22112 | 7113 | 7 | 2354 | 263.5 | 6.24 |
| VrLEA-35 | [vigra.Vradi11g00450](https://legumeinfo.org/feature/Vigna/radiata/gene/Vradi11g00450.Vradi.ver6) | LEA-2 | 11 | 424428 | 425004 | 576 | 576 | 1 | 192 | 21.71 | 8.93 |
| VrLEA-36 | [vigra.Vradi11g01510](https://legumeinfo.org/feature/Vigna/radiata/gene/Vradi11g01510.Vradi.ver6) | LEA-2 | 11 | 1374050 | 1375049 | 999 | 426 | 1 | 142 | 15.18 | 8.54 |
| VrLEA-37 | [vigra.Vradi11g01890](https://legumeinfo.org/feature/Vigna/radiata/gene/Vradi11g01890.Vradi.ver6) | LEA-2 | 11 | 1725901 | 1726522 | 621 | 621 | 1 | 207 | 23.4 | 9.11 |
| VrLEA-38 | [vigra.Vradi11g01900](https://legumeinfo.org/feature/Vigna/radiata/gene/Vradi11g01900.Vradi.ver6) | LEA-2 | 11 | 1735501 | 1736018 | 517 | 447 | 2 | 149 | 16.48 | 9.34 |
| VrLEA-39 | [vigra.Vradi11g07680](https://legumeinfo.org/feature/Vigna/radiata/gene/Vradi11g07680.Vradi.ver6) | LEA-2 | 11 | 8001559 | 8002039 | 480 | 480 | 1 | 160 | 17.83 | 8.56 |
| VrLEA-40 | [vigra.Vradi0083s00030](https://legumeinfo.org/feature/Vigna/radiata/gene/Vradi0083s00030.Vradi.ver6) | LEA-3 | vigra.scaffold_83 | 915994 | 916276 | 282 | 282 | 1 | 94 | 10.25 | 9.16 |
| VrLEA-41 | [vigra.Vradi03g06090](https://legumeinfo.org/feature/Vigna/radiata/gene/Vradi03g06090.Vradi.ver6) | LEA-3 | 3 | 7541305 | 7541698 | 393 | 312 | 2 | 104 | 10.95 | 8.95 |
| VrLEA-42 | [vigra.Vradi06g11560](https://legumeinfo.org/feature/Vigna/radiata/gene/Vradi06g11560.Vradi.ver6) | LEA-3 | 6 | 27937283 | 27937780 | 497 | 321 | 2 | 107 | 11.58 | 4.65 |
| VrLEA-43 | [vigra.Vradi07g25150](https://legumeinfo.org/feature/Vigna/radiata/gene/Vradi07g25150.Vradi.ver6) | LEA-3 | 7 | 48516968 | 48517342 | 374 | 288 | 2 | 96 | 10.43 | 9.65 |
| VrLEA-44 | [vigra.Vradi08g10310](https://legumeinfo.org/feature/Vigna/radiata/gene/Vradi08g10310.Vradi.ver6) | LEA-3 | 8 | 28125636 | 28129638 | 4002 | 603 | 2 | 201 | 22.66 | 8.38 |
| VrLEA-45 | [vigra.Vradi08g20160](https://legumeinfo.org/feature/Vigna/radiata/gene/Vradi08g20160.Vradi.ver6) | LEA-3 | 8 | 42259973 | 42260451 | 478 | 288 | 2 | 96 | 10.53 | 7.15 |
| VrLEA-46 | [vigra.Vradi03g04050](https://legumeinfo.org/feature/Vigna/radiata/gene/Vradi03g04050.Vradi.ver6) | LEA-4 | 3 | 5538641 | 5539647 | 1006 | 921 | 2 | 307 | 33.58 | 6.93 |
| VrLEA-47 | [vigra.Vradi08g06040](https://legumeinfo.org/feature/Vigna/radiata/gene/Vradi08g06040.Vradi.ver6) | LEA-4 | 8 | 13671717 | 13673582 | 1865 | 1560 | 3 | 520 | 55.96 | 5.39 |
| VrLEA-48 | [vigra.Vradi04g00330](https://legumeinfo.org/feature/Vigna/radiata/gene/Vradi04g00330.Vradi.ver6) | LEA-5 | 4 | 891101 | 891621 | 520 | 291 | 2 | 97 | 10.22 | 8.58 |
| VrLEA-49 | [vigra.Vradi09g09460](https://legumeinfo.org/feature/Vigna/radiata/gene/Vradi09g09460.Vradi.ver6) | LEA-5 | 9 | 19292625 | 19293164 | 539 | 300 | 2 | 100 | 10.87 | 5.81 |
| VrLEA-50 | [vigra.Vradi0374s00060](https://legumeinfo.org/feature/Vigna/radiata/gene/Vradi0374s00060.Vradi.ver6) | SMP | vigra.scaffold_374 | 35501 | 36381 | 880 | 798 | 2 | 266 | 27.17 | 4.47 |
| VrLEA-51 | [vigra.Vradi05g17820](https://legumeinfo.org/feature/Vigna/radiata/gene/Vradi05g17820.Vradi.ver6) | SMP | 5 | 27214309 | 27215407 | 1098 | 486 | 2 | 162 | 16.53 | 4.78 |
| VrLEA-52 | [vigra.Vradi08g09340](https://legumeinfo.org/feature/Vigna/radiata/gene/Vradi08g09340.Vradi.ver6) | SMP | 8 | 26042284 | 26043125 | 841 | 774 | 2 | 258 | 26.72 | 4.97 |
| VrLEA-53 | [vigra.Vradi08g13000](https://legumeinfo.org/feature/Vigna/radiata/gene/Vradi08g13000.Vradi.ver6) | SMP | 8 | 32608450 | 32609977 | 1527 | 1021 | 4 | 268 | 27.9 | 5.29 |
| VrLEA-54 | [vigra.Vradi09g02740](https://legumeinfo.org/feature/Vigna/radiata/gene/Vradi09g02740.Vradi.ver6) | SMP | 9 | 3015477 | 3015900 | 423 | 423 | 1 | 141 | 14.92 | 8.44 |
| VrLEA-55 | [vigra.Vradi05g15040](https://legumeinfo.org/feature/Vigna/radiata/gene/Vradi05g15040.Vradi.ver6) | DHN | 5 | 23963728 | 23964993 | 1265 | 1067 | 2 | 199 | 22.26 | 5.18 |

**Supplementary Table S2.** The *VrLEA* candidates identified in adzuki bean genome and their gene characteristics

| **Alias Name** | **Gene ID** | **Family** | **LG** | **Gene Start (bps)** | **Gene End (bps)** | **Genomic Length** | **CDS Length** | **Number of Exon** | **Protein length (aa)** | **Protein Mol. Wt (kDa)** | **pI** |
| --- | --- | --- | --- | --- | --- | --- | --- | --- | --- | --- | --- |
| VaLEA-1 | [Vigan.Vang0039ss01060](https://legumeinfo.org/feature/Vigna/angularis/gene/vigan.Gyeongwon.v3.Vang0039ss01060) | LEA-1 | Scaf_39 | 1750973 | 1756215 | 5242 | 789 | 1 | 263 | 30.20 | 9.18 |
| VaLEA-2 | [Vigan.Vang06g24290](https://legumeinfo.org/feature/Vigna/angularis/gene/vigan.Gyeongwon.v3.Vang06g24290) | LEA-1 | 6 | 34606123 | 34606980 | 857 | 624 | 2 | 208 | 21.21 | 4.86 |
| VaLEA-3 | [Vigan.Vang0002ss01070](https://legumeinfo.org/feature/Vigna/angularis/gene/vigan.Gyeongwon.v3.Vang0002ss01070) | LEA-2 | Scaf_2 | 1903850 | 1904330 | 480 | 480 | 1 | 160 | 17.82 | 8.5 |
| VaLEA-4 | [Vigan.Vang0011ss00170](https://legumeinfo.org/feature/Vigna/angularis/gene/vigan.Gyeongwon.v3.Vang0011ss00170) | LEA-2 | Scaf_11 | 298614 | 299271 | 657 | 657 | 1 | 219 | 24.40 | 9.14 |
| VaLEA-5 | [Vigan.Vang0017s00950](https://legumeinfo.org/feature/Vigna/angularis/gene/vigan.Gyeongwon.v3.Vang0017s00950) | LEA-2 | Scaf_17 | 1317762 | 1318518 | 756 | 756 | 1 | 252 | 27.52 | 8.64 |
| VaLEA-6 | [Vigan.Vang0021ss00160](https://legumeinfo.org/feature/Vigna/angularis/gene/vigan.Gyeongwon.v3.Vang0021ss00160) | LEA-2 | Scaf_21 | 236722 | 237466 | 744 | 744 | 1 | 248 | 27.25 | 9.8 |
| VaLEA-7 | [Vigan.Vang0032ss02040](https://legumeinfo.org/feature/Vigna/angularis/gene/vigan.Gyeongwon.v3.Vang0032ss02040) | LEA-2 | Scaf_32 | 3427142 | 3427703 | 561 | 561 | 1 | 187 | 20.27 | 6.15 |
| VaLEA-8 | [Vigan.Vang0032ss02200](https://legumeinfo.org/feature/Vigna/angularis/gene/vigan.Gyeongwon.v3.Vang0032ss02200) | LEA-2 | Scaf_32 | 3819848 | 3820472 | 624 | 624 | 1 | 208 | 23.22 | 9.63 |
| VaLEA-9 | [Vigan.Vang0045ss01510](https://legumeinfo.org/feature/Vigna/angularis/gene/vigan.Gyeongwon.v3.Vang0045ss01510) | LEA-2 | Scaf_45 | 1873401 | 1874142 | 741 | 741 | 1 | 247 | 27.27 | 8.46 |
| VaLEA-10 | [Vigan.Vang0058ss00220](https://legumeinfo.org/feature/Vigna/angularis/gene/vigan.Gyeongwon.v3.Vang0058ss00220) | LEA-2 | Scaf_58 | 239130 | 239883 | 753 | 753 | 1 | 251 | 27.29 | 9.59 |
| VaLEA-11 | [Vigan.Vang0071ss00150](https://legumeinfo.org/feature/Vigna/angularis/gene/vigan.Gyeongwon.v3.Vang0071ss00150) | LEA-2 | Scaf_71 | 176675 | 177428 | 753 | 753 | 1 | 251 | 27.44 | 7.66 |
| VaLEA-12 | [Vigan.Vang0084s00110](https://legumeinfo.org/feature/Vigna/angularis/gene/vigan.Gyeongwon.v3.Vang0084s00110) | LEA-2 | Scaf_84 | 103824 | 104457 | 633 | 633 | 1 | 211 | 23.13 | 9.05 |
| VaLEA-13 | [Vigan.Vang0159s00290](https://legumeinfo.org/feature/Vigna/angularis/gene/vigan.Gyeongwon.v3.Vang0159s00290) | LEA-2 | Scaf_159 | 318301 | 320044 | 1743 | 660 | 1 | 220 | 24.92 | 9.05 |
| VaLEA-14 | [Vigan.Vang0182s00520](https://legumeinfo.org/feature/Vigna/angularis/gene/vigan.Gyeongwon.v3.Vang0182s00520) | LEA-2 | Scaf_182 | 504329 | 505483 | 1154 | 720 | 3 | 240 | 26.77 | 9.12 |
| VaLEA-15 | [Vigan.Vang0182s00680](https://legumeinfo.org/feature/Vigna/angularis/gene/vigan.Gyeongwon.v3.Vang0182s00680) | LEA-2 | Scaf_182 | 591328 | 593525 | 2197 | 945 | 3 | 315 | 34.80 | 9.2 |
| VaLEA-16 | [Vigan.Vang01g00940](https://legumeinfo.org/feature/Vigna/angularis/gene/vigan.Gyeongwon.v3.Vang01g00940) | LEA-2 | 1 | 993893 | 994589 | 696 | 696 | 1 | 232 | 26.37 | 10.41 |
| VaLEA-17 | [Vigan.Vang01g04160](https://legumeinfo.org/feature/Vigna/angularis/gene/vigan.Gyeongwon.v3.Vang01g04160) | LEA-2 | 1 | 4977551 | 4980065 | 2514 | 1050 | 2 | 350 | 38.81 | 4.63 |
| VaLEA-18 | [Vigan.Vang01g06270](https://legumeinfo.org/feature/Vigna/angularis/gene/vigan.Gyeongwon.v3.Vang01g06270) | LEA-2 | 1 | 7890146 | 7891831 | 1685 | 831 | 1 | 277 | 30.64 | 8.72 |
| VaLEA-19 | [Vigan.Vang01g07860](https://legumeinfo.org/feature/Vigna/angularis/gene/vigan.Gyeongwon.v3.Vang01g07860) | LEA-2 | 1 | 10128780 | 10129569 | 789 | 789 | 1 | 263 | 29.53 | 9.16 |
| VaLEA-20 | [Vigan.Vang02g02100](https://legumeinfo.org/feature/Vigna/angularis/gene/vigan.Gyeongwon.v3.Vang02g02100) | LEA-2 | 2 | 2264116 | 2266636 | 2520 | 921 | 3 | 307 | 33.38 | 9.05 |
| VaLEA-21 | [Vigan.Vang03g05170](https://legumeinfo.org/feature/Vigna/angularis/gene/vigan.Gyeongwon.v3.Vang03g05170) | LEA-2 | 3 | 5191042 | 5221215 | 30173 | 3993 | 1 | 661 | 75.00 | 8.65 |
| VaLEA-22 | [Vigan.Vang03g09810](https://legumeinfo.org/feature/Vigna/angularis/gene/vigan.Gyeongwon.v3.Vang03g09810) | LEA-2 | 3 | 10320224 | 10325347 | 5123 | 1983 | 1 | 261 | 29.90 | 8.94 |
| VaLEA-23 | [Vigan.Vang0450s00030](https://legumeinfo.org/feature/Vigna/angularis/gene/vigan.Gyeongwon.v3.Vang0450s00030) | LEA-2 | Scaf_450 | 26065 | 35390 | 9325 | 966 | 1 | 184 | 19.96 | 6.6 |
| VaLEA-24 | [Vigan.Vang04g03970](https://legumeinfo.org/feature/Vigna/angularis/gene/vigan.Gyeongwon.v3.Vang04g03970) | LEA-2 | 4 | 5190313 | 5191509 | 1196 | 552 | 2 | 216 | 24.50 | 9.15 |
| VaLEA-25 | [Vigan.Vang04g05740](https://legumeinfo.org/feature/Vigna/angularis/gene/vigan.Gyeongwon.v3.Vang04g05740) | LEA-2 | 4 | 7454364 | 7455818 | 1454 | 648 | 1 | 422 | 46.98 | 5.33 |
| VaLEA-26 | [Vigan.Vang04g07650](https://legumeinfo.org/feature/Vigna/angularis/gene/vigan.Gyeongwon.v3.Vang04g07650) | LEA-2 | 4 | 10589885 | 10593508 | 3623 | 1266 | 1 | 232 | 25.33 | 8.47 |
| VaLEA-27 | [Vigan.Vang04g07710](https://legumeinfo.org/feature/Vigna/angularis/gene/vigan.Gyeongwon.v3.Vang04g07710) | LEA-2 | 4 | 10838570 | 10840148 | 1578 | 696 | 3 | 335 | 37.95 | 9.17 |
| VaLEA-28 | [Vigan.Vang05g05520](https://legumeinfo.org/feature/Vigna/angularis/gene/vigan.Gyeongwon.v3.Vang05g05520) | LEA-2 | 5 | 5148555 | 5150588 | 2033 | 1005 | 3 | 228 | 25.98 | 8.52 |
| VaLEA-29 | [Vigan.Vang06g02760](https://legumeinfo.org/feature/Vigna/angularis/gene/vigan.Gyeongwon.v3.Vang06g02760) | LEA-2 | 6 | 3045827 | 3046511 | 684 | 684 | 1 | 250 | 27.69 | 8.95 |
| VaLEA-30 | [Vigan.Vang06g11720](https://legumeinfo.org/feature/Vigna/angularis/gene/vigan.Gyeongwon.v3.Vang06g11720) | LEA-2 | 6 | 13575095 | 13575845 | 750 | 750 | 1 | 381 | 43.37 | 8.87 |
| VaLEA-31 | [Vigan.Vang06g25090](https://legumeinfo.org/feature/Vigna/angularis/gene/vigan.Gyeongwon.v3.Vang06g25090) | LEA-2 | 6 | 35956766 | 35967327 | 10561 | 1143 | 1 | 238 | 26.81 | 8.74 |
| VaLEA-32 | [Vigan.Vang06g25110](https://legumeinfo.org/feature/Vigna/angularis/gene/vigan.Gyeongwon.v3.Vang06g25110) | LEA-2 | 6 | 36002256 | 36002970 | 714 | 714 | 1 | 207 | 23.43 | 9.2 |
| VaLEA-33 | [Vigan.Vang07g06640](https://legumeinfo.org/feature/Vigna/angularis/gene/vigan.Gyeongwon.v3.Vang07g06640) | LEA-2 | 7 | 7549207 | 7555435 | 6228 | 621 | 1 | 202 | 21.93 | 8.71 |
| VaLEA-34 | [Vigan.Vang07g07120](https://legumeinfo.org/feature/Vigna/angularis/gene/vigan.Gyeongwon.v3.Vang07g07120) | LEA-2 | 7 | 7928944 | 7929550 | 606 | 606 | 1 | 483 | 53.21 | 8.98 |
| VaLEA-35 | [Vigan.Vang07g08020](https://legumeinfo.org/feature/Vigna/angularis/gene/vigan.Gyeongwon.v3.Vang07g08020) | LEA-2 | 7 | 8844535 | 8849118 | 4583 | 1449 | 1 | 194 | 22.12 | 9.04 |
| VaLEA-36 | [Vigan.Vang07g08250](https://legumeinfo.org/feature/Vigna/angularis/gene/vigan.Gyeongwon.v3.Vang07g08250) | LEA-2 | 7 | 9010779 | 9011361 | 582 | 582 | 1 | 314 | 34.09 | 9.41 |
| VaLEA-37 | [Vigan.Vang0869s00040](https://legumeinfo.org/feature/Vigna/angularis/gene/vigan.Gyeongwon.v3.Vang0869s00040) | LEA-2 | Scaf_869 | 6405 | 10527 | 4122 | 942 | 2 | 220 | 24.44 | 9.75 |
| VaLEA-38 | [Vigan.Vang08g00860](https://legumeinfo.org/feature/Vigna/angularis/gene/vigan.Gyeongwon.v3.Vang08g00860) | LEA-2 | 8 | 1507321 | 1507981 | 660 | 660 | 1 | 153 | 16.87 | 5.68 |
| VaLEA-39 | [Vigan.Vang08g00880](https://legumeinfo.org/feature/Vigna/angularis/gene/vigan.Gyeongwon.v3.Vang08g00880) | LEA-2 | 8 | 1518366 | 1519826 | 1460 | 459 | 2 | 282 | 31.00 | 4.97 |
| VaLEA-40 | [Vigan.Vang0973s00010](https://legumeinfo.org/feature/Vigna/angularis/gene/vigan.Gyeongwon.v3.Vang0973s00010) | LEA-2 | Scaf_973 | 48104 | 50203 | 2099 | 846 | 2 | 187 | 20.62 | 7.58 |
| VaLEA-41 | [Vigan.Vang09g02100](https://legumeinfo.org/feature/Vigna/angularis/gene/vigan.Gyeongwon.v3.Vang09g02100) | LEA-2 | 9 | 3718026 | 3718587 | 561 | 561 | 1 | 330 | 36.93 | 9.72 |
| VaLEA-42 | [Vigan.Vang10g00610](https://legumeinfo.org/feature/Vigna/angularis/gene/vigan.Gyeongwon.v3.Vang10g00610) | LEA-2 | 10 | 565241 | 566797 | 1556 | 990 | 3 | 159 | 17.19 | 6.25 |
| VaLEA-43 | [Vigan.Vang10g05920](https://legumeinfo.org/feature/Vigna/angularis/gene/vigan.Gyeongwon.v3.Vang10g05920) | LEA-2 | 10 | 6236163 | 6238616 | 2453 | 447 | 1 | 224 | 24.61 | 8.02 |
| VaLEA-44 | [Vigan.Vang10g06350](https://legumeinfo.org/feature/Vigna/angularis/gene/vigan.Gyeongwon.v3.Vang10g06350) | LEA-2 | 10 | 6646593 | 6647265 | 672 | 672 | 1 | 204 | 22.20 | 8 |
| VaLEA-45 | [Vigan.Vang11g00760](https://legumeinfo.org/feature/Vigna/angularis/gene/vigan.Gyeongwon.v3.Vang11g00760) | LEA-2 | 11 | 968948 | 969560 | 612 | 612 | 1 | 356 | 39.43 | 9.41 |
| VaLEA-46 | [Vigan.Vang11g03960](https://legumeinfo.org/feature/Vigna/angularis/gene/vigan.Gyeongwon.v3.Vang11g03960) | LEA-2 | 11 | 5800096 | 5801584 | 1488 | 1068 | 3 | 322 | 35.71 | 9.5 |
| VaLEA-47 | [Vigan.Vang1709s00010](https://legumeinfo.org/feature/Vigna/angularis/gene/vigan.Gyeongwon.v3.Vang1709s00010) | LEA-2 | Scaf_1709 | 146 | 2369 | 2223 | 966 | 3 | 98 | 10.92 | 8.59 |
| VaLEA-48 | [Vigan.Vang0027ss00890](https://legumeinfo.org/feature/Vigna/angularis/gene/vigan.Gyeongwon.v3.Vang0027ss00890) | LEA-3 | Scaf_27 | 760366 | 760660 | 294 | 294 | 1 | 98 | 10.90 | 6.74 |
| VaLEA-49 | [Vigan.Vang0206s00320](https://legumeinfo.org/feature/Vigna/angularis/gene/vigan.Gyeongwon.v3.Vang0206s00320) | LEA-3 | Scaf_206 | 599812 | 600106 | 294 | 294 | 1 | 89 | 10.35 | 9.06 |
| VaLEA-50 | [Vigan.Vang0273s00310](https://legumeinfo.org/feature/Vigna/angularis/gene/vigan.Gyeongwon.v3.Vang0273s00310) | LEA-3 | Scaf_273 | 474142 | 474882 | 740 | 267 | 2 | 131 | 14.47 | 8.82 |
| VaLEA-51 | [Vigan.Vang03g09940](https://legumeinfo.org/feature/Vigna/angularis/gene/vigan.Gyeongwon.v3.Vang03g09940) | LEA-3 | 3 | 10554642 | 10555531 | 889 | 393 | 2 | 138 | 15.12 | 8.48 |
| VaLEA-52 | [Vigan.Vang04g16360](https://legumeinfo.org/feature/Vigna/angularis/gene/vigan.Gyeongwon.v3.Vang04g16360) | LEA-3 | 4 | 25880464 | 25882065 | 1601 | 414 | 2 | 147 | 16.52 | 7.21 |
| VaLEA-53 | [Vigan.Vang04g16370](https://legumeinfo.org/feature/Vigna/angularis/gene/vigan.Gyeongwon.v3.Vang04g16370) | LEA-3 | 4 | 25877141 | 25877696 | 555 | 441 | 2 | 108 | 11.79 | 8.89 |
| VaLEA-54 | [Vigan.Vang06g18980](https://legumeinfo.org/feature/Vigna/angularis/gene/vigan.Gyeongwon.v3.Vang06g18980) | LEA-3 | 6 | 24440174 | 24440915 | 741 | 324 | 3 | 114 | 12.00 | 11.02 |
| VaLEA-55 | [Vigan.Vang09g08100](https://legumeinfo.org/feature/Vigna/angularis/gene/vigan.Gyeongwon.v3.Vang09g08100) | LEA-3 | 9 | 12231940 | 12232743 | 803 | 342 | 2 | 83 | 9.36 | 7.7 |
| VaLEA-56 | [Vigan.Vang11g00410](https://legumeinfo.org/feature/Vigna/angularis/gene/vigan.Gyeongwon.v3.Vang11g00410) | LEA-3 | 11 | 454651 | 454900 | 249 | 249 | 1 | 108 | 11.78 | 9.21 |
| VaLEA-57 | [Vigan.Vang11g17100](https://legumeinfo.org/feature/Vigna/angularis/gene/vigan.Gyeongwon.v3.Vang11g17100) | LEA-3 | 11 | 22788709 | 22789567 | 858 | 324 | 2 | 307 | 33.55 | 6.34 |
| VaLEA-58 | [Vigan.Vang0134s00340](https://legumeinfo.org/feature/Vigna/angularis/gene/vigan.Gyeongwon.v3.Vang0134s00340) | LEA-4 | Scaf_134 | 543669 | 544675 | 1006 | 921 | 2 | 495 | 53.28 | 5.55 |
| VaLEA-59 | [Vigan.Vang06g20240](https://legumeinfo.org/feature/Vigna/angularis/gene/vigan.Gyeongwon.v3.Vang06g20240) | LEA-4 | 6 | 26995828 | 26997666 | 1838 | 1485 | 3 | 97 | 1063.00 | 8.02 |
| VaLEA-60 | [Vigan.Vang0022ss01990](https://legumeinfo.org/feature/Vigna/angularis/gene/vigan.Gyeongwon.v3.Vang0022ss01990) | SMP | Scaf_22 | 3024086 | 3025807 | 1721 | 558 | 2 | 258 | 26.00 | 4.4 |
| VaLEA-61 | [Vigan.Vang06g04580](https://legumeinfo.org/feature/Vigna/angularis/gene/vigan.Gyeongwon.v3.Vang06g04580) | SMP | 6 | 4953193 | 4954073 | 880 | 774 | 2 | 259 | 26.88 | 5.6 |
| VaLEA-62 | [Vigan.Vang06g10390](https://legumeinfo.org/feature/Vigna/angularis/gene/vigan.Gyeongwon.v3.Vang06g10390) | DHN | 6 | 12144569 | 12145950 | 1381 | 777 | 4 | 256 | 26.31 | 4.68 |
| VaLEA-63 | [Vigan.Vang06g16990](https://legumeinfo.org/feature/Vigna/angularis/gene/vigan.Gyeongwon.v3.Vang06g16990) | DHN | 6 | 20751651 | 20752578 | 927 | 768 | 2 | 253 | 26.11 | 5.62 |
| VaLEA-64 | [Vigan.Vang02g04810](https://legumeinfo.org/feature/Vigna/angularis/gene/vigan.Gyeongwon.v3.Vang02g04810) | DHN | 2 | 6426514 | 6427273 | 759 | 759 | 1 | 1050 | 116.30 | 6.55 |

**Supplementary Table S3.** The *VrLEA* candidates identified in cowpea genome and their gene characteristics

| **Alias Name** | **Gene ID** | **Family** | **LG** | **Gene Start (bps)** | **Gene End (bps)** | **Genomic Length** | **CDS Length** | **Number of Exon** | **Protein length (aa)** | **Protein Mol. Wt (kDa)** | **pI** |
| --- | --- | --- | --- | --- | --- | --- | --- | --- | --- | --- | --- |
| VuLEA-1 | [vigun.Vigun01g016300](https://legumeinfo.org/feature/Vigna/unguiculata/gene/vigun.IT97K-499-35.gnm1.ann1.Vigun01g016300) | LEA-1 | 1 | 1773560 | 1774766 | 1206 | 538 | 2 | 64 | 43.74 | 4.26 |
| VuLEA-2 | [vigun.Vigun01g124100](https://legumeinfo.org/feature/Vigna/unguiculata/gene/vigun.IT97K-499-35.gnm1.ann1.Vigun01g124100) | LEA-1 | 1 | 30102574 | 30103307 | 733 | 644 | 2 | 111 | 52.39 | 4.17 |
| VuLEA-3 | [vigun.Vigun01g124200](https://legumeinfo.org/feature/Vigna/unguiculata/gene/vigun.IT97K-499-35.gnm1.ann1.Vigun01g124200) | LEA-1 | 1 | 30107015 | 30107904 | 889 | 809 | 2 | 163 | 65.41 | 4.1 |
| VuLEA-4 | [vigun.Vigun03g292500](https://legumeinfo.org/feature/Vigna/unguiculata/gene/vigun.IT97K-499-35.gnm1.ann1.Vigun03g292500) | LEA-1 | 3 | 47756395 | 47757106 | 711 | 624 | 2 | 117 | 51.44 | 4.15 |
| VuLEA-5 | [vigun.Vigun07g025800](https://legumeinfo.org/feature/Vigna/unguiculata/gene/vigun.IT97K-499-35.gnm1.ann1.Vigun07g025800) | LEA-1 | 7 | 2314523 | 2315615 | 1092 | 914 | 2 | 204 | 74.58 | 4.06 |
| VuLEA-6 | [vigun.Vigun01g179000](https://legumeinfo.org/feature/Vigna/unguiculata/gene/vigun.IT97K-499-35.gnm1.ann1.Vigun01g179000) | LEA-2 | 1 | 35977226 | 35977892 | 666 | 666 | 1 | 222 | 55.99 | 4.04 |
| VuLEA-7 | [vigun.Vigun01g179200](https://legumeinfo.org/feature/Vigna/unguiculata/gene/vigun.IT97K-499-35.gnm1.ann1.Vigun01g179200) | LEA-2 | 1 | 35985828 | 35987070 | 1242 | 1242 | 1 | 228 | 106.44 | 3.97 |
| VuLEA-8 | [vigun.Vigun01g179300](https://legumeinfo.org/feature/Vigna/unguiculata/gene/vigun.IT97K-499-35.gnm1.ann1.Vigun01g179300) | LEA-2 | 1 | 35994437 | 35995507 | 1070 | 1070 | 1 | 210 | 92.45 | 3.94 |
| VuLEA-9 | [vigun.Vigun02g107300](https://legumeinfo.org/feature/Vigna/unguiculata/gene/vigun.IT97K-499-35.gnm1.ann1.Vigun02g107300) | LEA-2 | 2 | 26123794 | 26124463 | 669 | 669 | 1 | 223 | 55.7 | 4.06 |
| VuLEA-10 | [vigun.Vigun02g179300](https://legumeinfo.org/feature/Vigna/unguiculata/gene/vigun.IT97K-499-35.gnm1.ann1.Vigun02g179300) | LEA-2 | 2 | 32043717 | 32044646 | 929 | 929 | 1 | 188 | 79.49 | 4.04 |
| VuLEA-11 | [vigun.Vigun02g179400](https://legumeinfo.org/feature/Vigna/unguiculata/gene/vigun.IT97K-499-35.gnm1.ann1.Vigun02g179400) | LEA-2 | 2 | 32052633 | 32053491 | 858 | 858 | 1 | 220 | 71.79 | 4.07 |
| VuLEA-12 | [vigun.Vigun02g184200](https://legumeinfo.org/feature/Vigna/unguiculata/gene/vigun.IT97K-499-35.gnm1.ann1.Vigun02g184200) | LEA-2 | 2 | 32408859 | 32409474 | 615 | 615 | 1 | 205 | 52.98 | 4.03 |
| VuLEA-13 | [vigun.Vigun02g196900](https://legumeinfo.org/feature/Vigna/unguiculata/gene/vigun.IT97K-499-35.gnm1.ann1.Vigun02g196900) | LEA-2 | 2 | 33202775 | 33203598 | 823 | 823 | 1 | 190 | 70.8 | 4.06 |
| VuLEA-14 | [vigun.Vigun02g198900](https://legumeinfo.org/feature/Vigna/unguiculata/gene/vigun.IT97K-499-35.gnm1.ann1.Vigun02g198900) | LEA-2 | 2 | 33384447 | 33385020 | 573 | 573 | 1 | 191 | 47.61 | 4.15 |
| VuLEA-15 | [vigun.Vigun03g007300](https://legumeinfo.org/feature/Vigna/unguiculata/gene/vigun.IT97K-499-35.gnm1.ann1.Vigun03g007300) | LEA-2 | 3 | 494596 | 496210 | 1614 | 1435 | 3 | 300 | 121.93 | 3.92 |
| VuLEA-16 | [vigun.Vigun03g040300](https://legumeinfo.org/feature/Vigna/unguiculata/gene/vigun.IT97K-499-35.gnm1.ann1.Vigun03g040300) | LEA-2 | 3 | 3076113 | 3077134 | 1021 | 1021 | 1 | 224 | 84.89 | 4.05 |
| VuLEA-17 | [vigun.Vigun03g075100](https://legumeinfo.org/feature/Vigna/unguiculata/gene/vigun.IT97K-499-35.gnm1.ann1.Vigun03g075100) | LEA-2 | 3 | 6245000 | 6246136 | 1136 | 1136 | 1 | 246 | 93.88 | 3.98 |
| VuLEA-18 | [vigun.Vigun03g217200](https://legumeinfo.org/feature/Vigna/unguiculata/gene/vigun.IT97K-499-35.gnm1.ann1.Vigun03g217200) | LEA-2 | 3 | 36165099 | 36165723 | 624 | 624 | 1 | 208 | 51.85 | 4.11 |
| VuLEA-19 | [vigun.Vigun03g278100](https://legumeinfo.org/feature/Vigna/unguiculata/gene/vigun.IT97K-499-35.gnm1.ann1.Vigun03g278100) | LEA-2 | 3 | 45568163 | 45569937 | 1774 | 1561 | 3 | 323 | 133.29 | 3.87 |
| VuLEA-20 | [vigun.Vigun04g012900](https://legumeinfo.org/feature/Vigna/unguiculata/gene/vigun.IT97K-499-35.gnm1.ann1.Vigun04g012900) | LEA-2 | 4 | 936209 | 938924 | 2715 | 1564 | 3 | 305 | 130.67 | 3.93 |
| VuLEA-21 | [vigun.Vigun04g028300](https://legumeinfo.org/feature/Vigna/unguiculata/gene/vigun.IT97K-499-35.gnm1.ann1.Vigun04g028300) | LEA-2 | 4 | 2203474 | 2205816 | 2342 | 1630 | 3 | 248 | 137.49 | 3.92 |
| VuLEA-22 | [vigun.Vigun04g033500](https://legumeinfo.org/feature/Vigna/unguiculata/gene/vigun.IT97K-499-35.gnm1.ann1.Vigun04g033500) | LEA-2 | 4 | 2776198 | 2776922 | 724 | 724 | 1 | 298 | 61.04 | 4.09 |
| VuLEA-23 | [vigun.Vigun04g033600](https://legumeinfo.org/feature/Vigna/unguiculata/gene/vigun.IT97K-499-35.gnm1.ann1.Vigun04g033600) | LEA-2 | 4 | 2781639 | 2782798 | 1159 | 1159 | 1 | 208 | 98.88 | 4 |
| VuLEA-24 | [vigun.Vigun05g060200](https://legumeinfo.org/feature/Vigna/unguiculata/gene/vigun.IT97K-499-35.gnm1.ann1.Vigun05g060200) | LEA-2 | 5 | 5154384 | 5157014 | 2630 | 2386 | 2 | 208 | 199.46 | 3.91 |
| VuLEA-25 | [vigun.Vigun05g080900](https://legumeinfo.org/feature/Vigna/unguiculata/gene/vigun.IT97K-499-35.gnm1.ann1.Vigun05g080900) | LEA-2 | 5 | 7623737 | 7626299 | 2562 | 2055 | 3 | 183 | 173.68 | 3.91 |
| VuLEA-26 | [vigun.Vigun05g081900](https://legumeinfo.org/feature/Vigna/unguiculata/gene/vigun.IT97K-499-35.gnm1.ann1.Vigun05g081900) | LEA-2 | 5 | 7714098 | 7716796 | 2698 | 1191 | 3 | 193 | 99.11 | 4.02 |
| VuLEA-27 | [vigun.Vigun05g082000](https://legumeinfo.org/feature/Vigna/unguiculata/gene/vigun.IT97K-499-35.gnm1.ann1.Vigun05g082000) | LEA-2 | 5 | 7719050 | 7720315 | 1265 | 1265 | 1 | 198 | 105.09 | 4.02 |
| VuLEA-28 | [vigun.Vigun05g216700](https://legumeinfo.org/feature/Vigna/unguiculata/gene/vigun.IT97K-499-35.gnm1.ann1.Vigun05g216700) | LEA-2 | 5 | 40858821 | 40859544 | 723 | 870 | 1 | 224 | 71.9 | 4.08 |
| VuLEA-29 | [vigun.Vigun05g232900](https://legumeinfo.org/feature/Vigna/unguiculata/gene/vigun.IT97K-499-35.gnm1.ann1.Vigun05g232900) | LEA-2 | 5 | 42687008 | 42688317 | 1309 | 1309 | 1 | 241 | 114.07 | 3.88 |
| VuLEA-30 | [vigun.Vigun05g233000](https://legumeinfo.org/feature/Vigna/unguiculata/gene/vigun.IT97K-499-35.gnm1.ann1.Vigun05g233000) | LEA-2 | 5 | 42697653 | 42698654 | 1001 | 1001 | 1 | 248 | 87.33 | 3.94 |
| VuLEA-31 | [vigun.Vigun05g275200](https://legumeinfo.org/feature/Vigna/unguiculata/gene/vigun.IT97K-499-35.gnm1.ann1.Vigun05g275200) | LEA-2 | 5 | 46543923 | 46545351 | 1428 | 1428 | 1 | 221 | 123.06 | 3.88 |
| VuLEA-32 | [vigun.Vigun05g284900](https://legumeinfo.org/feature/Vigna/unguiculata/gene/vigun.IT97K-499-35.gnm1.ann1.Vigun05g284900) | LEA-2 | 5 | 47263448 | 47266016 | 2568 | 1623 | 3 | 252 | 136.48 | 3.95 |
| VuLEA-33 | [vigun.Vigun05g286100](https://legumeinfo.org/feature/Vigna/unguiculata/gene/vigun.IT97K-499-35.gnm1.ann1.Vigun05g286100) | LEA-2 | 5 | 47348780 | 47350497 | 1717 | 1380 | 3 | 310 | 113.78 | 3.98 |
| VuLEA-34 | [vigun.Vigun06g013900](https://legumeinfo.org/feature/Vigna/unguiculata/gene/vigun.IT97K-499-35.gnm1.ann1.Vigun06g013900) | LEA-2 | 6 | 6537972 | 6538970 | 998 | 998 | 1 | 200 | 82.89 | 4.02 |
| VuLEA-35 | [vigun.Vigun06g022400](https://legumeinfo.org/feature/Vigna/unguiculata/gene/vigun.IT97K-499-35.gnm1.ann1.Vigun06g022400) | LEA-2 | 6 | 10497861 | 10500824 | 2963 | 1624 | 3 | 249 | 134.22 | 3.98 |
| VuLEA-36 | [vigun.Vigun06g066900](https://legumeinfo.org/feature/Vigna/unguiculata/gene/vigun.IT97K-499-35.gnm1.ann1.Vigun06g066900) | LEA-2 | 6 | 19544891 | 19545653 | 762 | 762 | 1 | 300 | 64.4 | 4.05 |
| VuLEA-37 | [vigun.Vigun06g092900](https://legumeinfo.org/feature/Vigna/unguiculata/gene/vigun.IT97K-499-35.gnm1.ann1.Vigun06g092900) | LEA-2 | 6 | 22507985 | 22509044 | 1059 | 1059 | 1 | 254 | 89.24 | 4.04 |
| VuLEA-38 | [vigun.Vigun06g210300](https://legumeinfo.org/feature/Vigna/unguiculata/gene/vigun.IT97K-499-35.gnm1.ann1.Vigun06g210300) | LEA-2 | 6 | 32339279 | 32340485 | 1206 | 1206 | 1 | 244 | 101.21 | 4.01 |
| VuLEA-39 | [vigun.Vigun06g229500](https://legumeinfo.org/feature/Vigna/unguiculata/gene/vigun.IT97K-499-35.gnm1.ann1.Vigun06g229500) | LEA-2 | 6 | 33657024 | 33660350 | 3326 | 1697 | 2 | 202 | 143.18 | 3.88 |
| VuLEA-40 | [vigun.Vigun07g013400](https://legumeinfo.org/feature/Vigna/unguiculata/gene/vigun.IT97K-499-35.gnm1.ann1.Vigun07g013400) | LEA-2 | 7 | 1216084 | 1216798 | 714 | 714 | 1 | 240 | 61 | 4.01 |
| VuLEA-41 | [vigun.Vigun07g013600](https://legumeinfo.org/feature/Vigna/unguiculata/gene/vigun.IT97K-499-35.gnm1.ann1.Vigun07g013600) | LEA-2 | 7 | 1223165 | 1225661 | 2496 | 699 | 1 | 238 | 58.44 | 4.05 |
| VuLEA-42 | [vigun.Vigun07g013700](https://legumeinfo.org/feature/Vigna/unguiculata/gene/vigun.IT97K-499-35.gnm1.ann1.Vigun07g013700) | LEA-2 | 7 | 1233076 | 1235021 | 1945 | 1945 | 1 | 233 | 165.83 | 3.86 |
| VuLEA-43 | [vigun.Vigun07g165200](https://legumeinfo.org/feature/Vigna/unguiculata/gene/vigun.IT97K-499-35.gnm1.ann1.Vigun07g165200) | LEA-2 | 7 | 27751293 | 27753008 | 1715 | 1530 | 2 | 210 | 128.4 | 3.95 |
| VuLEA-44 | [vigun.Vigun07g245300](https://legumeinfo.org/feature/Vigna/unguiculata/gene/vigun.IT97K-499-35.gnm1.ann1.Vigun07g245300) | LEA-2 | 7 | 36616797 | 36617956 | 1159 | 1159 | 1 | 255 | 98.42 | 3.97 |
| VuLEA-45 | [vigun.Vigun08g138600](https://legumeinfo.org/feature/Vigna/unguiculata/gene/vigun.IT97K-499-35.gnm1.ann1.Vigun08g138600) | LEA-2 | 8 | 31084421 | 31085805 | 1384 | 1384 | 1 | 228 | 116.66 | 3.98 |
| VuLEA-46 | [vigun.Vigun08g178200](https://legumeinfo.org/feature/Vigna/unguiculata/gene/vigun.IT97K-499-35.gnm1.ann1.Vigun08g178200) | LEA-2 | 8 | 34807297 | 34808396 | 1099 | 1099 | 1 | 263 | 91.82 | 3.98 |
| VuLEA-47 | [vigun.Vigun08g182400](https://legumeinfo.org/feature/Vigna/unguiculata/gene/vigun.IT97K-499-35.gnm1.ann1.Vigun08g182400) | LEA-2 | 8 | 35199009 | 35201202 | 2193 | 1333 | 3 | 274 | 109.42 | 4.04 |
| VuLEA-48 | [vigun.Vigun08g213200,](https://legumeinfo.org/feature/Vigna/unguiculata/gene/vigun.IT97K-499-35.gnm1.ann1.Vigun08g213200) | LEA-2 | 8 | 37429869 | 37431437 | 1568 | 1568 | 1 | 322 | 232132.8 | 3.94 |
| VuLEA-49 | [vigun.Vigun09g004600](https://legumeinfo.org/feature/Vigna/unguiculata/gene/vigun.IT97K-499-35.gnm1.ann1.Vigun09g004600) | LEA-2 | 9 | 348767 | 349361 | 594 | 594 | 1 | 198 | 49.4 | 4.09 |
| VuLEA-50 | [vigun.Vigun09g140200](https://legumeinfo.org/feature/Vigna/unguiculata/gene/vigun.IT97K-499-35.gnm1.ann1.Vigun09g140200) | LEA-2 | 9 | 30216379 | 30217012 | 633 | 633 | 1 | 211 | 54.43 | 4.07 |
| VuLEA-51 | [vigun.Vigun09g175100](https://legumeinfo.org/feature/Vigna/unguiculata/gene/vigun.IT97K-499-35.gnm1.ann1.Vigun09g175100) | LEA-2 | 9 | 34643209 | 34644289 | 1080 | 1080 | 1 | 241 | 91.61 | 3.99 |
| VuLEA-52 | [vigun.Vigun10g138300](https://legumeinfo.org/feature/Vigna/unguiculata/gene/vigun.IT97K-499-35.gnm1.ann1.Vigun10g138300) | LEA-2 | 10 | 35481001 | 35483120 | 2119 | 704 | 2 | 153 | 59.53 | 4.11 |
| VuLEA-53 | [vigun.Vigun10g138600](https://legumeinfo.org/feature/Vigna/unguiculata/gene/vigun.IT97K-499-35.gnm1.ann1.Vigun10g138600) | LEA-2 | 10 | 35493640 | 35495481 | 1841 | 1841 | 1 | 220 | 153.07 | 3.93 |
| VuLEA-54 | [vigun.Vigun10g152200](https://legumeinfo.org/feature/Vigna/unguiculata/gene/vigun.IT97K-499-35.gnm1.ann1.Vigun10g152200) | LEA-2 | 10 | 37211845 | 37213241 | 1396 | 1396 | 1 | 298 | 118.41 | 3.93 |
| VuLEA-55 | [vigun.Vigun10g173200](https://legumeinfo.org/feature/Vigna/unguiculata/gene/vigun.IT97K-499-35.gnm1.ann1.Vigun10g173200) | LEA-2 | 10 | 39176683 | 39177439 | 756 | 756 | 1 | 252 | 64.82 | 3.96 |
| VuLEA-56 | [vigun.Vigun11g111500](https://legumeinfo.org/feature/Vigna/unguiculata/gene/vigun.IT97K-499-35.gnm1.ann1.Vigun11g111500) | LEA-2 | 11 | 31334311 | 31334878 | 567 | 567 | 1 | 189 | 48.14 | 4.14 |
| VuLEA-57 | [vigun.Vigun11g115600](https://legumeinfo.org/feature/Vigna/unguiculata/gene/vigun.IT97K-499-35.gnm1.ann1.Vigun11g115600) | LEA-2 | 11 | 31976138 | 31977239 | 1101 | 1101 | 1 | 223 | 93.1 | 4.01 |
| VuLEA-58 | [vigun.Vigun11g122800](https://legumeinfo.org/feature/Vigna/unguiculata/gene/vigun.IT97K-499-35.gnm1.ann1.Vigun11g122800) | LEA-2 | 11 | 33011793 | 33012902 | 1109 | 1109 | 1 | 208 | 98.11 | 3.93 |
| VuLEA-59 | [vigun.Vigun11g126000](https://legumeinfo.org/feature/Vigna/unguiculata/gene/vigun.IT97K-499-35.gnm1.ann1.Vigun11g126000) | LEA-2 | 11 | 33326975 | 33328905 | 1930 | 1930 | 1 | 187 | 164.4 | 3.88 |
| VuLEA-60 | [vigun.Vigun11g198900](https://legumeinfo.org/feature/Vigna/unguiculata/gene/vigun.IT97K-499-35.gnm1.ann1.Vigun11g198900) | LEA-2 | 11 | 39701649 | 39704474 | 2825 | 1819 | 4 | 315 | 154.16 | 3.85 |
| VuLEA-61 | [vigun.Vigun01g192300](https://legumeinfo.org/feature/Vigna/unguiculata/gene/vigun.IT97K-499-35.gnm1.ann1.Vigun01g192300) | LEA-3 | 1 | 37036808 | 37037218 | 410 | 294 | 2 | 98 | 24.77 | 4.3 |
| VuLEA-62 | [vigun.Vigun01g192400](https://legumeinfo.org/feature/Vigna/unguiculata/gene/vigun.IT97K-499-35.gnm1.ann1.Vigun01g192400) | LEA-3 | 1 | 37040056 | 37040787 | 731 | 621 | 2 | 107 | 52.77 | 4.13 |
| VuLEA-63 | [vigun.Vigun03g421400](https://legumeinfo.org/feature/Vigna/unguiculata/gene/vigun.IT97K-499-35.gnm1.ann1.Vigun03g421400) | LEA-3 | 3 | 62713750 | 62714769 | 1019 | 932 | 2 | 108 | 78.42 | 4.08 |
| VuLEA-64 | [vigun.Vigun06g118800](https://legumeinfo.org/feature/Vigna/unguiculata/gene/vigun.IT97K-499-35.gnm1.ann1.Vigun06g118800) | LEA-3 | 6 | 24699882 | 24700967 | 1085 | 999 | 2 | 95 | 85.64 | 4.03 |
| VuLEA-65 | [vigun.Vigun07g120900](https://legumeinfo.org/feature/Vigna/unguiculata/gene/vigun.IT97K-499-35.gnm1.ann1.Vigun07g120900) | LEA-3 | 7 | 22339268 | 22340409 | 1141 | 923 | 2 | 191 | 78.41 | 4.04 |
| VuLEA-66 | [vigun.Vigun07g254100](https://legumeinfo.org/feature/Vigna/unguiculata/gene/vigun.IT97K-499-35.gnm1.ann1.Vigun07g254100) | LEA-3 | 7 | 37254713 | 37256167 | 1454 | 1323 | 2 | 104 | 111.4 | 3.98 |
| VuLEA-67 | [vigun.Vigun09g013000](https://legumeinfo.org/feature/Vigna/unguiculata/gene/vigun.IT97K-499-35.gnm1.ann1.Vigun09g013000) | LEA-3 | 9 | 971820 | 973222 | 1402 | 1248 | 2 | 91 | 106.07 | 4.02 |
| VuLEA-68 | [vigun.Vigun10g157900](https://legumeinfo.org/feature/Vigna/unguiculata/gene/vigun.IT97K-499-35.gnm1.ann1.Vigun10g157900) | LEA-3 | 10 | 37729284 | 37730467 | 1183 | 426 | 2 | 95 | 35.65 | 4.22 |
| VuLEA-69 | [vigun.Vigun01g167600](https://legumeinfo.org/feature/Vigna/unguiculata/gene/vigun.IT97K-499-35.gnm1.ann1.Vigun01g167600) | LEA-4 | 1 | 34938179 | 34939479 | 1300 | 1221 | 2 | 305 | 95.74 | 4.02 |
| VuLEA-70 | [vigun.Vigun03g407000](https://legumeinfo.org/feature/Vigna/unguiculata/gene/vigun.IT97K-499-35.gnm1.ann1.Vigun03g407000) | LEA-4 | 3 | 61436993 | 61441704 | 4711 | 1909 | 2 | 374 | 155.66 | 3.9 |
| VuLEA-71 | [vigun.Vigun07g064700](https://legumeinfo.org/feature/Vigna/unguiculata/gene/vigun.IT97K-499-35.gnm1.ann1.Vigun07g064700) | LEA-4 | 7 | 7539284 | 7541576 | 2292 | 2186 | 2 | 557 | 171.34 | 3.9 |
| VuLEA-72 | [vigun.Vigun05g098500](https://legumeinfo.org/feature/Vigna/unguiculata/gene/vigun.IT97K-499-35.gnm1.ann1.Vigun05g098500) | LEA-5 | 5 | 9646730 | 9647692 | 962 | 800 | 2 | 113 | 63.8 | 4.19 |
| VuLEA-73 | [vigun.Vigun10g081800](https://legumeinfo.org/feature/Vigna/unguiculata/gene/vigun.IT97K-499-35.gnm1.ann1.Vigun10g081800) | LEA-5 | 10 | 23028839 | 23030023 | 1184 | 1006 | 2 | 100 | 82.08 | 4.06 |
| VuLEA-74 | [vigun.Vigun03g284300](https://legumeinfo.org/feature/Vigna/unguiculata/gene/vigun.IT97K-499-35.gnm1.ann1.Vigun03g284300) | LEA-6 | 3 | 46521280 | 46521832 | 552 | 552 | 1 | 88 | 46.26 | 4.19 |
| VuLEA-75 | [vigun.Vigun03g008500](https://legumeinfo.org/feature/Vigna/unguiculata/gene/vigun.IT97K-499-35.gnm1.ann1.Vigun03g008500) | DEHYDRIN | 3 | 580120 | 580762 | 642 | 642 | 1 | 92 | 53 | 4.2 |
| VuLEA-76 | [vigun.Vigun04g077600](https://legumeinfo.org/feature/Vigna/unguiculata/gene/vigun.IT97K-499-35.gnm1.ann1.Vigun04g077600) | DEHYDRIN | 4 | 10636979 | 10637804 | 825 | 270 | 1 | 119 | 21.11 | 4.37 |
| VuLEA-77 | [vigun.Vigun09g267800](https://legumeinfo.org/feature/Vigna/unguiculata/gene/vigun.IT97K-499-35.gnm1.ann1.Vigun09g267800) | DEHYDRIN | 9 | 43090298 | 43091223 | 925 | 825 | 2 | 181 | 68.54 | 4.06 |
| VuLEA-78 | [vigun.Vigun09g268600](https://legumeinfo.org/feature/Vigna/unguiculata/gene/vigun.IT97K-499-35.gnm1.ann1.Vigun09g268600) | DEHYDRIN | 9 | 43134285 | 43135515 | 1230 | 1028 | 2 | 201 | 83 | 4.1 |
| VuLEA-79 | [vigun.Vigun03g008500](https://legumeinfo.org/feature/Vigna/unguiculata/gene/vigun.IT97K-499-35.gnm1.ann1.Vigun03g008500) | SMP | 3 | 580120 | 580762 | 642 | 740 | 2 | 190 | 60.06 | 4.08 |
| VuLEA-80 | [vigun.Vigun04g077600](https://legumeinfo.org/feature/Vigna/unguiculata/gene/vigun.IT97K-499-35.gnm1.ann1.Vigun04g077600) | SMP | 4 | 10636979 | 10637804 | 825 | 1307 | 3 | 261 | 107.02 | 3.95 |
| VuLEA-81 | [vigun.Vigun09g267800](https://legumeinfo.org/feature/Vigna/unguiculata/gene/vigun.IT97K-499-35.gnm1.ann1.Vigun09g267800) | SMP | 9 | 43090298 | 43091223 | 925 | 1396 | 3 | 278 | 114.02 | 3.95 |
| VuLEA-82 | [vigun.Vigun09g268600](https://legumeinfo.org/feature/Vigna/unguiculata/gene/vigun.IT97K-499-35.gnm1.ann1.Vigun09g268600) | SMP | 9 | 43134285 | 43135515 | 1230 | 1284 | 2 |  | 104.79 | 3.97 |

**Supplementary Table S4.** Gene specific primers developed from *VrLEA* gene family and their functional annotation with *Vigna glabrescens*

| **Primer Code** | **Primer Sequence** | **Start** | **End** | **Tm** | **GC** | **Product Size (bp)** | **Accession no.** | **Annotation** |
| --- | --- | --- | --- | --- | --- | --- | --- | --- |
| VrLEA-1 | CCTCGGACAGAAACAACCGA | 262 | 281 | 59.97 | 55.00 | 128 | [KU708229.1](https://www.ncbi.nlm.nih.gov/nucleotide/KU708229.1?report=genbank&log$=nucltop&blast_rank=1&RID=HX682YR7013) | [*Vigna glabrescens* ATP synthase beta subunit (atpB) gene](https://blast.ncbi.nlm.nih.gov/Blast.cgi) |
|  | GACCCGGTACTGAGGGGTAT | 389 | 370 | 60.11 | 60.00 |  |  |  |
| VrLEA-2 | CTCCATTCTTGCAGCCAGGT | 128 | 147 | 60.32 | 55.00 | 113 | [KX087877.1](https://www.ncbi.nlm.nih.gov/nucleotide/KX087877.1?report=genbank&log$=nucltop&blast_rank=1&RID=HX6AFWTH013) | [*Vigna glabrescens* isolate 029 maturase K (matK) gene](https://blast.ncbi.nlm.nih.gov/Blast.cgi) |
|  | CCCAACATGCTGGTCTTCCA | 240 | 221 | 60.25 | 55.00 |  |  |  |
| VrLEA-3 | CAGGTCAAGACCATCGTTGC | 28 | 47 | 59.2 | 55.00 | 117 | [KX087877.1](https://www.ncbi.nlm.nih.gov/nucleotide/KX087877.1?report=genbank&log$=nucltop&blast_rank=1&RID=HX6D2GVV01R) | *Vigna glabrescens* isolate 029 maturase K (matK) gene |
|  | ACGTTTGGGTGCATTGGGTA | 144 | 125 | 60.18 | 50.00 |  |  |  |
| VrLEA-4 | GGTGGTTTGCTCACCTGAAG | 44 | 63 | 59.05 | 55.00 | 185 | [KX087722.1](https://www.ncbi.nlm.nih.gov/nucleotide/KX087722.1?report=genbank&log$=nucltop&blast_rank=1&RID=HX6GC8H1016) | *Vigna glabrescens* isolate 030 5.8S ribosomal RNA gene |
|  | CTGCCCTTGGTAGCTGATGT | 228 | 209 | 59.75 | 55.00 |  |  |  |
| VrLEA-5 | ACCAGCTTTTCCTTAGCGTT | 6 | 25 | 57.73 | 45.00 | 162 | [JN008219.1](https://www.ncbi.nlm.nih.gov/nucleotide/JN008219.1?report=genbank&log$=nucltop&blast_rank=1&RID=HX6JB7HX013) | *Vigna glabrescens* bio-material DNA 1240 trnK gene, partial sequence; and maturase K (matK) gene |
|  | CTGTCTGGTTTTTGCGATGGA | 167 | 147 | 59.12 | 47.62 |  |  |  |
| VrLEA-6 | ACTGCAGTGGCAGTAATGGT | 67 | 86 | 59.6 | 50.00 | 102 | [KX087877.1](https://www.ncbi.nlm.nih.gov/nucleotide/KX087877.1?report=genbank&log$=nucltop&blast_rank=1&RID=HX6NFJ53016) | *Vigna glabrescens* isolate 029 maturase K (matK) gene |
|  | TGAGGTGTGTCGTGTGAGAC | 168 | 149 | 59.62 | 55.00 |  |  |  |
| VrLEA-7 | ACCTGGGAGTTTTTCAGTCTTGT | 1024 | 1046 | 60.05 | 43.48 | 114 | [KX087564.1](https://www.ncbi.nlm.nih.gov/nucleotide/KX087564.1?report=genbank&log$=nucltop&blast_rank=1&RID=HX6RWVTK01R) | igna glabrescens isolate 028 PsbA (psbA) gene, partial cds; and psbA-trnH intergenic spacer |
|  | AAAATCCGGGTGTGCACTGT | 1137 | 1118 | 60.47 | 50.00 |  |  |  |
| VrLEA-8 | TCTTCGTCATCTCACTCGATTC | 2 | 23 | 57.72 | 45.45 | 140 | [KX087722.1](https://www.ncbi.nlm.nih.gov/nucleotide/KX087722.1?report=genbank&log$=nucltop&blast_rank=1&RID=HX6TVJJF016) | *Vigna glabrescens* isolate 030 5.8S ribosomal RNA gene, partial sequence; internal transcribed spacer 2 |
|  | CAGAGTCTGTTTTCGCGTGC | 141 | 122 | 60.11 | 55.00 |  |  |  |
| VrLEA-9 | ACTGATGTCTCCCTCACACA | 16 | 35 | 57.98 | 50.00 | 175 | [KX087722.1](https://www.ncbi.nlm.nih.gov/nucleotide/KX087722.1?report=genbank&log$=nucltop&blast_rank=1&RID=HX6VKT7B016) | *Vigna glabrescens* isolate 030 5.8S ribosomal RNA gene, partial sequence; internal transcribed spacer 2 |
|  | AGGGTGTCAAGTTTACCCGA | 190 | 171 | 58.57 | 50.00 |  |  |  |
| VrLEA-10 | CGGCGTCATCCTCATCGTTA | 24 | 43 | 59.97 | 55.00 | 175 | [KX087877.1](https://www.ncbi.nlm.nih.gov/nucleotide/KX087877.1?report=genbank&log$=nucltop&blast_rank=1&RID=HX6XG92G01R) | *Vigna glabrescens* isolate 029 maturase K (matK) gene |
|  | GGCGTTGTAGGTGATGGTCA | 198 | 179 | 60.04 | 55.00 |  |  |  |
| VrLEA-11 | GGAGGAGCCGAACTTTCCAA | 685 | 704 | 59.96 | 55.00 | 141 | [KX087409.1](https://www.ncbi.nlm.nih.gov/nucleotide/KX087409.1?report=genbank&log$=nucltop&blast_rank=1&RID=GDCT3KZ2013) | *Vigna glabrescens* isolate 029 ribulose-1,5-bisphosphate carboxylase/oxygenase large subunit (rbcL) gene |
|  | TGATAACTGATCTGTCCCCCG | 825 | 805 | 58.96 | 52.38 |  |  |  |
| VrLEA-12 | CAGTAAGCCAACGGGACGAA | 1 | 20 | 60.32 | 55.00 | 177 | [KX087877.1](https://www.ncbi.nlm.nih.gov/nucleotide/KX087877.1?report=genbank&log$=nuclalign&blast_rank=1&RID=GDD5VZ7N013) | *Vigna glabrescens* isolate 029 maturase K (matK) gene |
|  | GGTTGGAAGGGCTCTGAACA | 177 | 158 | 59.89 | 55.00 |  |  |  |
| VrLEA-13 | TCGCCCTTCCTCTACGGTAA | 370 | 389 | 60.03 | 55.00 | 184 | KX087877.1 | *Vigna glabrescens* isolate 029 maturase K (matK) gene |
|  | AGAGAACCATGGTCAAGTGC | 553 | 534 | 57.81 | 50.00 |  |  |  |
| VrLEA-14 | CACCCAATTCAAGCAGTGCC | 260 | 279 | 60.04 | 55.00 | 194 | KU708229.1 | *Vigna glabrescens* ATP synthase beta subunit (atpB) gene |
|  | TGTCTGAATGTCCGCAGTGG | 453 | 434 | 60.32 | 55.00 |  |  |  |
| VrLEA-15 | TGATTGGCCACAGGCATTCT | 168 | 187 | 59.96 | 50.00 | 170 | JN008219.1 | *Vigna glabrescens* bio-material DNA 1240 trnK gene, partial sequence; and maturase K (matK) gene |
|  | TCTTGGAATTGAGGTTATCTGTGT | 337 | 314 | 57.87 | 37.50 |  |  |  |
| VrLEA-16 | CTCAAGCCAACCAAGCCAAC | 124 | 143 | 59.97 | 55.00 | 158 | KU708229.1 | *Vigna glabrescens* ATP synthase beta subunit (atpB) gene |
|  | TAGGTGCTGAGACGGTCGTA | 281 | 262 | 60.04 | 55.00 |  |  |  |
| VrLEA-17 | GTGTAATGGTCACTGGGGCA | 113 | 132 | 59.86 | 55.00 | 126 | KX087877.1 | *Vigna glabrescens* isolate 029 maturase K (matK) gene |
|  | CCTCCAAAGTGGCAGACACA | 238 | 219 | 59.29 | 50.00 |  |  |  |
| VrLEA-18 | TGAGCTTGGCAAGGGACATT | 597 | 616 | 59.89 | 50.00 | 159 | KX087722 | *Vigna glabrescens* isolate 030 5.8S ribosomal RNA gene, partial sequence; internal transcribed spacer 2, complete sequence; and 28S ribosomal RNA gene |
|  | TGAAGTAACTCCACACGTCGG | 755 | 735 | 60.00 | 52.38 |  |  |  |
| VrLEA-19 | CAGGCATCGATTTGAGCACG | 86 | 105 | 59.97 | 55.00 | 128 | Y19408 | *Vigna glabrescens* DNA for internal transcribed spacer 1 |
|  | TGACGTTTTCGCCTGCTTTG | 213 | 194 | 59.97 | 50.00 |  |  |  |
| VrLEA-20 | AGGACGTGGCCAAAGGTATC | 685 | 704 | 59.75 | 55.00 | 174 | KU708229 | *Vigna glabrescens* ATP synthase beta subunit (atpB) gene |
|  | GCTTCTGGGGACCCTCGT | 858 | 841 | 61.00 | 66.67 |  |  |  |
| VrLEA-21 | AACCATTGCCCTGAATCCCT | 1356 | 1375 | 59.29 | 50.00 | 183 | [KX087877.1](https://www.ncbi.nlm.nih.gov/nucleotide/KX087877.1?report=genbank&log$=nucltop&blast_rank=1&RID=HX6ZS15N016) | *Vigna glabrescens* isolate 029 maturase K (matK) gene |
|  | ACGTTCTCCATAAACCGACTCA | 1538 | 1517 | 59.44 | 45.45 |  |  |  |
| VrLEA-22 | GCATTGTGGGTGAAGTTCCG | 272 | 291 | 59.76 | 55.00 | 158 | [KU708229.1](https://www.ncbi.nlm.nih.gov/nucleotide/KU708229.1?report=genbank&log$=nucltop&blast_rank=1&RID=HX71S2P1013) | *Vigna glabrescens* ATP synthase beta subunit (atpB) gene |
|  | CAGTGCAGCTGTTGATGTGA | 435 | 416 | 58.77 | 50.00 |  |  |  |
| VrLEA-23 | TGCTTCAGTTTGGCTTGTTTTC | 1236 | 1257 | 58.48 | 40.91 | 170 | [JN008219.1](https://www.ncbi.nlm.nih.gov/nucleotide/JN008219.1?report=genbank&log$=nucltop&blast_rank=1&RID=HX73NPU801R) | *Vigna glabrescens* bio-material DNA 1240 trnK gene, partial sequence; and maturase K (matK) gene |
|  | ACAAAACTCCACCTCACAAACA | 1405 | 1384 | 58.57 | 40.91 |  |  |  |
| VrLEA-24 | TCCTCTGTCGTTCCACAAGC | 73 | 92 | 59.97 | 55.00 | 101 | [KX087409.1](https://www.ncbi.nlm.nih.gov/nucleotide/KX087409.1?report=genbank&log$=nucltop&blast_rank=1&RID=HX75A4DJ016) | *Vigna glabrescens* isolate 029 ribulose-1,5-bisphosphate carboxylase/oxygenase large subunit (rbcL) gene |
|  | TTCGGAGCTTGATCCTTGGG | 173 | 154 | 59.75 | 55.00 |  |  |  |
| VrLEA-25 | AGGTGCCAAAGAACAGCTCA | 350 | 369 | 59.82 | 50.00 | 137 | [KX087409.1](https://www.ncbi.nlm.nih.gov/nucleotide/KX087409.1?report=genbank&log$=nucltop&blast_rank=1&RID=HX77D4FF016) | *Vigna glabrescens* isolate 029 ribulose-1,5-bisphosphate carboxylase/oxygenase large subunit (rbcL) gene |
|  | TGCAGCCCCTGTGAAATCAA | 486 | 467 | 60.18 | 50.00 |  |  |  |
| VrLEA-26 | GCCCCACCAAGTACGCTAAT | 20 | 39 | 60.11 | 55.00 | 181 | [JN008219.1](https://www.ncbi.nlm.nih.gov/nucleotide/JN008219.1?report=genbank&log$=nucltop&blast_rank=1&RID=HX79CCMJ016) | *Vigna glabrescens* bio-material DNA 1240 trnK gene, partial sequence; and maturase K (matK) gene |
|  | CCACGTTGTCGTTGGGTTTC | 200 | 181 | 59.97 | 55.00 |  |  |  |
| VrLEA-27 | GTGGGCTTCAACTATGGGCT | 274 | 293 | 60.03 | 55.00 | 153 | [KU708229.1](https://www.ncbi.nlm.nih.gov/nucleotide/KU708229.1?report=genbank&log$=nucltop&blast_rank=1&RID=HX7BAXG001R) | *Vigna glabrescens* ATP synthase beta subunit (atpB) gene |
|  | GGATCCATCGGTTACGGCAA | 426 | 407 | 60.18 | 55.00 |  |  |  |
| VrLEA-28 | CGGCGTCTACTACGACCAAA | 144 | 163 | 59.83 | 55.00 | 172 | [KX087564.1](https://www.ncbi.nlm.nih.gov/nucleotide/KX087564.1?report=genbank&log$=nucltop&blast_rank=1&RID=HX7D2JV601R) | *Vigna glabrescens* isolate 028 PsbA (psbA) gene, partial cds; and psbA-trnH intergenic spacer |
|  | ACAAGAACTTGAACCTTGCTCAG | 315 | 293 | 59.37 | 43.48 |  |  |  |
| VrLEA-29 | GAAGAGCACGAACCGTCTGA | 414 | 433 | 60.04 | 55.00 | 189 | [KU708229.1](https://www.ncbi.nlm.nih.gov/nucleotide/KU708229.1?report=genbank&log$=nucltop&blast_rank=1&RID=HX7GYZ3M013) | *Vigna glabrescens* ATP synthase beta subunit (atpB) gene |
|  | CATTTTGCCTTCGCCTTCGT | 602 | 583 | 59.76 | 50.00 |  |  |  |
| VrLEA-30 | CCACCACTGCTACGTTCTGT | 121 | 140 | 59.97 | 55.00 | 129 | [AY962479.1](https://www.ncbi.nlm.nih.gov/nucleotide/AY962479.1?report=genbank&log$=nucltop&blast_rank=1&RID=HX7JYKNC013) | *Vigna glabrescens* voucher NI532 5.8S ribosomal RNA gene |
|  | TCCAACAATTGCAGCCAGGA | 249 | 230 | 60.18 | 50.00 |  |  |  |
| VrLEA-31 | AGCGACCAAGCAGTGAAGAA | 44 | 63 | 59.89 | 50.00 | 161 | [KU708229.1](https://www.ncbi.nlm.nih.gov/nucleotide/KU708229.1?report=genbank&log$=nucltop&blast_rank=1&RID=HX7MY37U013) | *Vigna glabrescens* ATP synthase beta subunit (atpB) gene |
|  | CACAGGGTCCTTGACACGAA | 204 | 185 | 59.89 | 55.00 |  |  |  |
| VrLEA-32 | GGAGTACTTGGTGAAGTCAAAGG | 117 | 139 | 58.93 | 47.83 | 188 | [KX087877.1](https://www.ncbi.nlm.nih.gov/nucleotide/KX087877.1?report=genbank&log$=nucltop&blast_rank=1&RID=HX7PME0P013) | *Vigna glabrescens* isolate 029 maturase K (matK) gene |
|  | CTCCGGTGTGAGAGAGAGGA | 304 | 285 | 60.03 | 55.00 |  |  |  |
| VrLEA-33 | TCGGTGGATCAGCTGAGGAT | 268 | 287 | 60.40 | 55.00 | 180 | [KX087722.1](https://www.ncbi.nlm.nih.gov/nucleotide/KX087722.1?report=genbank&log$=nucltop&blast_rank=1&RID=HX7SJTST01R) | *Vigna glabrescens* isolate 030 5.8S ribosomal RNA gene, partial sequence; internal transcribed spacer 2 |
|  | TGGCAAAGACCCTTCACACA | 447 | 428 | 59.74 | 50.00 |  |  |  |
| VrLEA-34 | TCAGCTGTGGTGTTGATGCT | 4417 | 4436 | 59.89 | 50.00 | 189 | [KU708229.1](https://www.ncbi.nlm.nih.gov/nucleotide/KU708229.1?report=genbank&log$=nucltop&blast_rank=1&RID=HX7UTJX301R) | *Vigna glabrescens* ATP synthase beta subunit (atpB) gene |
|  | TTGCGTTGGGGTTAAGGTGT | 4605 | 4586 | 60.11 | 50.00 |  |  |  |
| VrLEA-35 | ACCCCAACAAGGACTCAAGC | 188 | 207 | 60.18 | 55.00 | 128 | [JN008219.1](https://www.ncbi.nlm.nih.gov/nucleotide/JN008219.1?report=genbank&log$=nucltop&blast_rank=1&RID=HX7WXS94013) | *Vigna glabrescens* bio-material DNA 1240 trnK gene, partial sequence; and maturase K (matK) gene |
|  | ATCCCGGGTGTTGCTAGTTG | 315 | 296 | 60.04 | 55.00 |  |  |  |
| VrLEA-36 | TGTCTGGTAAAGAAGCCATCCC | 2 | 23 | 60.03 | 50.00 | 163 | [KU708229.1](https://www.ncbi.nlm.nih.gov/nucleotide/KU708229.1?report=genbank&log$=nucltop&blast_rank=1&RID=HX7ZK8MG016) | *Vigna glabrescens* ATP synthase beta subunit (atpB) gene |
|  | AACATGAAGCCGACCTGTGT | 164 | 145 | 59.89 | 50.00 |  |  |  |
| VrLEA-37 | CTTCGATTACGAGGCGAGCA | 306 | 325 | 60.25 | 55.00 | 185 | [KX087877.1](https://www.ncbi.nlm.nih.gov/nucleotide/KX087877.1?report=genbank&log$=nucltop&blast_rank=1&RID=HX81HUU5013) | *Vigna glabrescens* isolate 029 maturase K (matK) gene |
|  | GTTTGAGCATGCCTGGGTTG | 490 | 471 | 60.04 | 55.00 |  |  |  |
| VrLEA-38 | ATAGCAGTGTGAGCGTGCTT | 266 | 285 | 60.04 | 50.00 | 172 | [KU708229.1](https://www.ncbi.nlm.nih.gov/nucleotide/KU708229.1?report=genbank&log$=nucltop&blast_rank=1&RID=HX83PHA501R) | *Vigna glabrescens* ATP synthase beta subunit (atpB) gene |
|  | AGCCATTTGAATGGTCTCCCT | 437 | 417 | 59.36 | 47.62 |  |  |  |
| VrLEA-39 | TGATCCACCCGAGAATCCCT | 5 | 24 | 60.03 | 55.00 | 196 | [KX087564.1](https://www.ncbi.nlm.nih.gov/nucleotide/KX087564.1?report=genbank&log$=nucltop&blast_rank=1&RID=HX86519Z013) | *Vigna glabrescens* isolate 028 PsbA (psbA) gene, partial cds; and psbA-trnH intergenic spacer |
|  | ACCATCCTTGCTATCGGCTG | 200 | 181 | 59.89 | 55.00 |  |  |  |
| VrLEA-40 | AGACGCGGATACTCTGTTGC | 73 | 92 | 60.18 | 55.00 | 103 | [AY884259.1](https://www.ncbi.nlm.nih.gov/nucleotide/AY884259.1?report=genbank&log$=nucltop&blast_rank=1&RID=HX87VS8R013) | *Vigna glabrescens* 5S ribosomal RNA gene |
|  | AAACCTCGTACGTCGACACC | 175 | 156 | 60.04 | 55.00 |  |  |  |
| VrLEA-41 | ATTGCAACAACGTCAGGGGA | 139 | 158 | 60.18 | 50.00 | 123 | [JN008219.1](https://www.ncbi.nlm.nih.gov/nucleotide/JN008219.1?report=genbank&log$=nucltop&blast_rank=1&RID=HX89KMVX013) | *Vigna glabrescens* bio-material DNA 1240 trnK gene, partial sequence; and maturase K (matK) gene |
|  | GCGCAATTCAGCGACATCAA | 261 | 242 | 60.18 | 50.00 |  |  |  |
| VrLEA-42 | CTGCGGAAATGGCAACACAA | 143 | 162 | 59.97 | 50.00 | 132 | [KX087877.1](https://www.ncbi.nlm.nih.gov/nucleotide/KX087877.1?report=genbank&log$=nucltop&blast_rank=1&RID=HX8BK4HK013) | *Vigna glabrescens* isolate 029 maturase K (matK) gene |
|  | AACCAGTCACAGGGTTTGGG | 274 | 255 | 60.11 | 55.00 |  |  |  |
| VrLEA-43 | ATCGGCGAGGTTATGCAGTT | 53 | 72 | 59.82 | 50.00 | 118 | [JN008219.1](https://www.ncbi.nlm.nih.gov/nucleotide/JN008219.1?report=genbank&log$=nucltop&blast_rank=1&RID=HX8DHUKK013) | *Vigna glabrescens* bio-material DNA 1240 trnK gene, partial sequence; and maturase K (matK) gene |
|  | CCATCTCTTGTCACCGGCTT | 170 | 151 | 60.04 | 55.00 |  |  |  |
| VrLEA-44 | AGGAACAAGCTTTCCGCAGT | 85 | 104 | 60.18 | 50.00 | 136 | [KX087877.1](https://www.ncbi.nlm.nih.gov/nucleotide/KX087877.1?report=genbank&log$=nucltop&blast_rank=1&RID=HX8H0KHV01R) | *Vigna glabrescens* isolate 029 maturase K (matK) gene |
|  | TTTCACCGCCTCTCACCTTC | 220 | 201 | 59.97 | 55.00 |  |  |  |
| VrLEA-45 | GTTGCTTCTGGGTGGACTGA | 33 | 52 | 59.89 | 55.00 | 137 | [KX087722.1](https://www.ncbi.nlm.nih.gov/nucleotide/KX087722.1?report=genbank&log$=nucltop&blast_rank=1&RID=HX8K1ATS01R) | *Vigna glabrescens* isolate 030 5.8S ribosomal RNA gene, partial sequence; internal transcribed spacer 2 |
|  | AAATTCCTGTGCGAGGGTGT | 169 | 150 | 59.89 | 50.00 |  |  |  |
| VrLEA-46 | AGCAAGGTGGGTCGAAGATG | 371 | 390 | 60.04 | 55.00 | 108 | [KU708229.1](https://www.ncbi.nlm.nih.gov/nucleotide/KU708229.1?report=genbank&log$=nucltop&blast_rank=1&RID=HX8SA3KZ013) | *Vigna glabrescens* ATP synthase beta subunit (atpB) gene |
|  | TCGCAAAGTCCGTGTACTCC | 478 | 459 | 60.04 | 55.00 |  |  |  |
| VrLEA-47 | GAAAGTCGACCAGGGGCAAT | 1423 | 1442 | 60.32 | 55.00 | 112 | [AY963637.1](https://www.ncbi.nlm.nih.gov/nucleotide/AY963637.1?report=genbank&log$=nucltop&blast_rank=1&RID=HX8URVCE013) | *Vigna glabrescens* cultivar NI532 internal transcribed spacer 1 |
|  | TCACTACATGAACGACCCCC | 1534 | 1515 | 59.10 | 55.00 |  |  |  |
| VrLEA-48 | AACTGTGGTTCCTGGAAGGAG | 57 | 77 | 59.58 | 52.38 | 174 | [KX087409.1](https://www.ncbi.nlm.nih.gov/nucleotide/KX087409.1?report=genbank&log$=nucltop&blast_rank=1&RID=HX8XPFH5013) | *Vigna glabrescens* isolate 029 ribulose-1,5-bisphosphate carboxylase/oxygenase large subunit (rbcL) gene |
|  | CCAGACTTGTCCATGGTGCT | 230 | 211 | 59.96 | 55.00 |  |  |  |
| VrLEA-49 | GGGAGAGACTGTTGTTCCCG | 51 | 70 | 60.04 | 60.00 | 145 | [KX087722.1](https://www.ncbi.nlm.nih.gov/nucleotide/KX087722.1?report=genbank&log$=nucltop&blast_rank=1&RID=HX908EZT016) | *Vigna glabrescens* isolate 030 5.8S ribosomal RNA gene, partial sequence; internal transcribed spacer 2 |
|  | CCCACCCTTTCGTCCCATTT | 195 | 176 | 60.25 | 55.00 |  |  |  |
| VrLEA-50 | ACTCAGAACGAACAGGCTGG | 196 | 215 | 59.97 | 55.00 | 146 | [KU708229.1](https://www.ncbi.nlm.nih.gov/nucleotide/KU708229.1?report=genbank&log$=nucltop&blast_rank=1&RID=HX92Y28U013) | *Vigna glabrescens* ATP synthase beta subunit (atpB) gene |
|  | AAAAATAAAAACAGAACCTTGCCG | 341 | 318 | 57.40 | 33.33 |  |  |  |
| VrLEA-51 | CGACCCCATAACCATCGGAG | 132 | 151 | 59.97 | 60.00 | 193 | [KX087722.1](https://www.ncbi.nlm.nih.gov/nucleotide/KX087722.1?report=genbank&log$=nucltop&blast_rank=1&RID=HX95CB9M013) | *Vigna glabrescens* isolate 030 5.8S ribosomal RNA gene, partial sequence; internal transcribed spacer 2 |
|  | CTCTGTGGCATCGTGACTGT | 324 | 305 | 60.04 | 55.00 |  |  |  |
| VrLEA-52 | CCAGTCAGCAGCCACACATA | 543 | 562 | 60.04 | 55.00 | 185 | [KX087409.1](https://www.ncbi.nlm.nih.gov/nucleotide/KX087409.1?report=genbank&log$=nucltop&blast_rank=1&RID=HX97D5MM013) | *Vigna glabrescens* isolate 029 ribulose-1,5-bisphosphate carboxylase/oxygenase large subunit (rbcL) gene |
|  | CCCCACCAGGGGTAGTTTTC | 727 | 708 | 59.96 | 60.00 |  |  |  |
| VrLEA-53 | GGAGACCGATGGTGAACAGG | 322 | 341 | 60.11 | 60.00 | 200 | [KX087409.1](https://www.ncbi.nlm.nih.gov/nucleotide/KX087409.1?report=genbank&log$=nucltop&blast_rank=1&RID=HX9A58MX016) | *Vigna glabrescens* isolate 029 ribulose-1,5-bisphosphate carboxylase/oxygenase large subunit (rbcL) gene |
|  | TCTTTCCTGCCGTGAGAACC | 521 | 502 | 59.97 | 55.00 |  |  |  |
| VrLEA-54 | CAATCTCCTCCGTGCTCTGG | 190 | 209 | 60.18 | 60.00 | 120 | [KU708229.1](https://www.ncbi.nlm.nih.gov/nucleotide/KU708229.1?report=genbank&log$=nucltop&blast_rank=1&RID=HX9C32UU013) | *Vigna glabrescens* ATP synthase beta subunit (atpB) gene |
|  | GGTGTCTTTGTTGACGCCAC | 309 | 290 | 59.97 | 55.00 |  |  |  |
| VrLEA-55 | CATGGCAGAGGAAAGCCAGA | 112 | 131 | 60.03 | 55.00 | 128 | [JN008219.1](https://www.ncbi.nlm.nih.gov/nucleotide/JN008219.1?report=genbank&log$=nucltop&blast_rank=1&RID=HX9E9TNE016) | *Vigna glabrescens* bio-material DNA 1240 trnK gene, partial sequence; and maturase K (matK) gene |
|  | CGATCACCTCTTCCTGAGGC | 239 | 220 | 59.90 | 60.00 |  |  |  |

**Supplementary Table S5.** List of primers used for qPCR analysis

| **Primer code** | **Family** | **Sequence** |
| --- | --- | --- |
| VrLEA1-1-F | LEA-1 | CCTCGGACAGAAACAACCGA |
| VrLEA1-1-R |  | GACCCGGTACTGAGGGGTAT |
| VrLEA2-2-F | LEA-2 | CTCCATTCTTGCAGCCAGGT |
| VrLEA2-2-R |  | CCCAACATGCTGGTCTTCCA |
| VrLEA3-40-F | LEA-3 | AGACGCGGATACTCTGTTGC |
| VrLEA3-40-R |  | AAACCTCGTACGTCGACACC |
| VrLEA4-47-F | LEA-4 | GAAAGTCGACCAGGGGCAAT |
| VrLEA4-47-R |  | TCACTACATGAACGACCCCC |
| VrLEA5-48-F | LEA-5 | AACTGTGGTTCCTGGAAGGAG |
| VrLEA5-48-R |  | CCAGACTTGTCCATGGTGCT |
| VrLEA-SMP--54-F | SMP | CAATCTCCTCCGTGCTCTGG |
| VrLEA-SMP-54-R |  | GGTGTCTTTGTTGACGCCAC |
| VrLEA-DHN-55-F | DHN | CATGGCAGAGGAAAGCCAGA |
| VrLEA-DHN-55-R |  | CGATCACCTCTTCCTGAGGC |
